# Managing Artificially Drained Low-Gradient Agricultural Headwaters for Enhanced Ecosystem Functions 

**DOI:** 10.3390/biology1030794

**Published:** 2012-12-10

**Authors:** Samuel C. Pierce, Robert Kröger, Reza Pezeshki

**Affiliations:** 1 Department of Wildlife, Fisheries, and Aquaculture, Mississippi State University, Starkville, MS 39762, USA; E-Mail: rkroger@cfr.msstate.edu; 2 Department of Biological Sciences, University of Memphis, Memphis, TN 38152, USA; E-Mail: pezeshki@memphis.edu

**Keywords:** channelization, eutrophication, restoration, wetland, ditch, stream, agroecology, drainage, nonpoint source pollution

## Abstract

Large tracts of lowlands have been drained to expand extensive agriculture into areas that were historically categorized as wasteland. This expansion in agriculture necessarily coincided with changes in ecosystem structure, biodiversity, and nutrient cycling. These changes have impacted not only the landscapes in which they occurred, but also larger water bodies receiving runoff from drained land. New approaches must append current efforts toward land conservation and restoration, as the continuing impacts to receiving waters is an issue of major environmental concern. One of these approaches is agricultural drainage management. This article reviews how this approach differs from traditional conservation efforts, the specific practices of drainage management and the current state of knowledge on the ecology of drainage ditches. A bottom-up approach is utilized, examining the effects of stochastic hydrology and anthropogenic disturbance on primary production and diversity of primary producers, with special regard given to how management can affect establishment of macrophytes and how macrophytes in agricultural landscapes alter their environment in ways that can serve to mitigate non-point source pollution and promote biodiversity in receiving waters.

## 1. Overview and Scope

Increases in agricultural productivity have resulted in widespread changes to the landscape that has significantly altered the functioning of aquatic ecosystem processes [1,2,3,4,5]. The recognition of this dysfunction has led to strategies to rehabilitate functionality of aquatic ecosystems to increase the services they provide [6,7,8,9,10,11,12,13,14,15]. This review is intended to provide an overview of research examining the ecological impacts of these changes and the resultant mitigation efforts currently utilized in surface waters associated with agricultural landscapes. Agriculture is ubiquitous, thus any synopsis of its environmental effects must rely upon some degree of generalization and some degree of abridgement. Likewise, the present assessment of management practices for reducing these impacts is in some ways limited. This treatment focuses specifically on those practices that alter drainage patterns in agricultural landscapes, both with regard to environmental impacts and amelioration of those impacts. Changes in hydrology and nonpoint source pollutant loads in surface waters, specifically sediment, nitrogen, and phosphorus, are discussed in detail and related to their effects on macroscopic aquatic organisms. Conversely, other important nonpoint source aquatic pollutants such as pesticides, metals, and pathogens are not discussed. General crop and livestock management practices, as well as the indirect effects of agriculture on the spread of non-native organisms, though pertinent, do not fall within the scope of this review. 

As stated in the title, the ecosystem of focus is the low-gradient, agricultural headwater network. A focus specifically on the interface between agricultural inputs and the aquatic environment, must inevitably examine drainage ditches that are either completely artificial, or have been altered to the extent that they more closely resemble a man-made ditch than a naturally occurring drainage feature. Such a system may be labeled ditch, canal, stream, or creek depending upon historic condition and degree of naturalization or anthropogenic disturbance. In the present review, the term stream, although inclusive of ditches, generally refers to systems that are assumed to be created and maintained by fluvial processes unless the context dictates otherwise. “Ditch” is used to describe systems either created or maintained by human activities in order to increase water conveyance; whereas “drainage” refers to the practice water removal, or, when used in conjunction with “network” or “system,” describes the entirety of streams and ditches modified for water conveyance.

As described by Davies *et al.* [16], low-gradient ditches are linear, angular and often have little relationship with natural landscape contours. This definition is still somewhat broad for the purposes of this review, as it describes structures ranging in size from in-field trenches or swales to canals that are effectively channelized rivers [17,18]. The former are essentially terrestrial systems, whereas the latter cannot be considered headwaters, and require a different management approach. This review specifically examines those systems that can broadly be defined as wadeable streams or linear wetlands, that is, those that experience a degree of inundation resulting in hydric soils, but that would be considered, at most, a 3^rd^ order stream [19]. Following the Strahler stream order designation, primary ditches are those that receive the majority of their inflow directly from agricultural fields; whereas higher order ditches are fed by both primary ditches and the surrounding landscape.

## 2. Impacts of Agricultural Expansion

As human society settles into a new millennium, in addition to looking ahead, a certain amount of retrospection can be expected regarding the role of our species in creating an environment conducive to maintaining a population of nearly seven billion. One important aspect of this retrospection is a consideration of how the achievements resulting in the population explosion of the 20^th^ century may have adversely affected the current and future well-being of our species, and the myriad other organisms comprising the ecosphere. Advances in agriculture and water management were fundamental for the exponential population growth of the last century and also for the improved quality of life experienced by many. Advances in communication, data processing, and environmental monitoring, however, have resulted in a qualitative shift in how we understand the relationship between technology and societal progress.

The availability of academic search engines on the internet allows a rudimentary quantification of this shift with respect to research publications. A Scopus search (scopus.com) for research and review articles published from January 2000 to August 2012 containing the words agriculture and either “environmental impact” or “ecological impact” resulted in 20,826 documents. This number of documents is indicative of the widespread knowledge among researchers that such impacts do exists. Note that a number of similar words could be substituted for impact, potentially increasing the documents returned. Limiting the search to occurrences only found in the title, abstract, or keyword list decreased the number of documents found by Scopus to 3,478. Over the twelve year period examined, research output on this topic more than doubled from 165 publications in 2000, to 365 in 2011, with over 195 publications catalogued midway through 2012.

Further limiting the search to only keywords and adding the additional requirement that drainage, ditch, or channelization appear in the keyword list further decreased the number of returned documents to 70, with 36% originating in the United States, and Australia and China accounting for another 10% each. A keyword search for (aquatic OR “water quality” OR pollution) AND agriculture AND (drainage OR ditch OR channelization) resulted in 400 publications. Over the period examined, the annual number of publications increased from eight in 2000, to 52 in 2011 and 32 documents catalogued by the middle of 2012. This greater than six-fold increase demonstrates the increasing concern among researchers that our aquatic resources are imperiled by current agricultural and water management practices, especially if one considers that the topic only produced an annual mean of 6.4 publications in the decade from 1989–1999. Approximately 40% of the publications originated in the United States, with Canada and the United Kingdom each comprising less than 8% of the total. Given this imbalance in research output related to the topic and inherent bias due the authors’ experiences, the present review is somewhat biased toward North America, specifically areas of extensive row crop agriculture in the lowlands of the Mississippi River Basin, referred to as the Mississippi Alluvial Valley (MAV). 

### 2.1. History of Wetland and Stream Losses to Agriculture

Agricultural drainage is associated with some of the earliest evidence of civilization. As cited by Beauchamp [20] and van Schilfgaarde [21], documents ditch construction in Mesopotamia as early as 9,000 years ago. Written evidence of the construction of drainage ditches for agriculture near the city of Memphis, Egypt, was recorded by Herodotus approximately 2,500 years ago [20]. Like the Pyramids of Giza, the drainage network at Memphis was reputedly ancient even then. Archaeological findings suggest that agricultural drainage may have been a common practice among early agricultural societies worldwide. Drainage ditches contemporaneous to the time of Herodotus discovered in the wetlands of Papua New Guinea have, in fact, been cited as evidence of prehistoric agriculture in the region [22,23,24], although this interpretation has been questioned [25]. 

**Figure 1:**
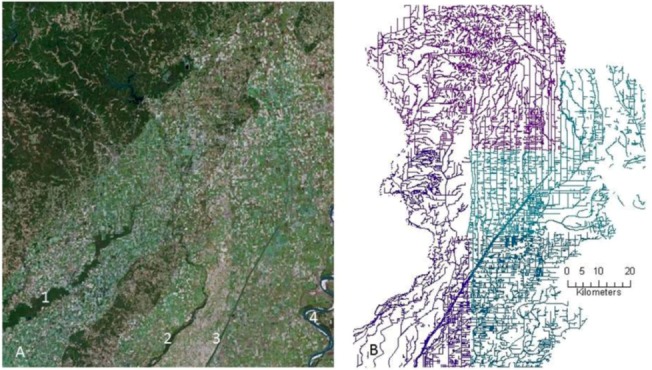
**(a)** Satellite photo showing the intersection of the Mississippi Alluvial valley and the eastern foothills of the Ozark Mountains on the border of Missouri and Arkansas, USA. The Black River (1, lower left) and the Saint Francis River (2, center) both have headwaters lying in the Ozark foothills at the top left, as well as in Crowley’s Ridge, the semi-forested area between the two rivers. The historic floodplains of their tributaries are clearly visible as non-forested pasture among the foothills. What remains of the Little River (3) is seen as a straight line between the Saint Francis River and the Mississippi River (4, bottom right corner). Much of the source water for both the Black and Saint Francis originates in closed canopy forest, and there is a high percentage of stream canopy coverage in the upland areas pastures. In contrast, the row cropping agriculture predominant in the lowlands creates a treeless landscape. The different colored specks are each individual fields bordered on at least two sides by a drainage ditch. **(b)** Stream diagram of the right half of Figure 1A, showing perennial streams between the Saint Francis River and the Mississippi River. The familiar dendritic and pinnate stream patterns at the top of the image changes to a trellis pattern of drainage ditches reflecting the change in topology and related increases in land use intensity.

The extent of agricultural drainage waxed and waned throughout history until technological advances in the 19^th^ and early 20^th^ century, including mechanization and subsurface drainage, led to a series of extensive drainage projects. In the MAV these projects reached truly monumental proportions. In southeastern Missouri alone, the Little River Drainage District, which began construction in 1914, moved almost 67 million cubic meters of earth, draining 176,000 hectares of land by its completion in 1929 (Figure 1, [20]). Following the devastating flooding of the Mississippi River in 1927, channelization and levee construction largely disconnected the river from its historic floodplain, allowing further agricultural encroachment, draining more than 20 million hectares in the mid-western region of the United States, which comprises a greater part of the Upper MAV [2,26]. Research on the impacts of extensive flood-control, channelization and ditching related to agriculture in the latter half of the 20^th^ century has sparked concerns over loss of wildlife habitat and changes in ecosystem functions. A recent review of the environmental impacts caused by agricultural drainage lists habitat loss or alteration, reduced water quality, and hydrologic alterations as the three greatest impacts [2]. Cumulative effects of these changes result in a pattern of increased disturbance, altered pathways of biogeochemical cycling, decreased habitat availability at small scales, and decreased habitat connectivity at larger scales [27,28,29,30].

The United States Environmental Protection Agency delineates between the proximate *cause* of aquatic impairment and the source of impairment. Agriculture is listed as the primary source of aquatic impairment for lotic systems [31], while it ranks third for open-water lentic [31] and wetlands systems [32]. Of the sites assessed, agriculture accounts for approximately 35% of lotic impairment, 18% of wetland impairment and 15% of open-water lentic impairment (Table 1). Given the extent of agricultural drainage features on the landscape, agriculture is undoubtedly indirectly affecting the hydrology of receiving waters in ways that have not been adequately quantified. The specific *causes* for impairment of continental waters related to agriculture in the Unites States are sedimentation in freshwater wetlands and lotic systems, and excess nutrients in open-water systems. 

More generally, the impact of extensive agriculture on aquatic systems is well documented in several regions including Europe [33,34] and China [35,36]. Another major source of impairment, hypoxia related to organic enrichment, can often be traced to agricultural inputs. Agriculturally-sourced organic enrichment is usually caused either by manure from organic fertilizer application and intensive livestock production [37], or from increased atmospheric carbon fixation due to eutrophication, as explained below, but may also be influenced by conservation management practices [38]. The cumulative effects of agricultural practices along headwaters can result in severe hypoxia in marine systems. For example, although the profundal zones of the Black Sea are well known for chronic hypoxia or anoxia, the highly productive fisheries of the northwestern continental shelf were decimated due to hypoxia that resulted in part from the cumulative effect of agricultural intensification along the tributaries of the Danube River [39].

**Table 1:** Causes and Sources of Impairment of US Waters related to agriculture and drainage modification. Adapted from EPA, 2002. See text in Section 2.1 for more recent rankings for streams and lakes (EPA 2009).

Header?			Rank of Cause of Impairment					Rank of Source of Impairment			
Water type	quantity assessed	% assessed	Nutrients	Sediment/ Siltation	Turbidity	Organic Enrichment /Low D.O.	Unknown	Agriculture	% Agriculture Impairment	Hydro-modification	Unknown
Rivers, Stream, Creeks **	1 x 10^6^ km	18.83	5	1	*	7	9	1	16	3	2
Lakes, Ponds, Reservoirs***	60,000 km^2^	36.53	1	4	10	3	*	2	14	5	1
Bays & estuaries	79,000 km^2^	34.85	2	*	7	3	5	*	*	8	1
Coastal shoreline	4,000 km	4.39	5	9	7	10	2	5	4.50	*	2
Oceans, Near coastal waters	12,800 km^2^	9.15	7	10	5	4	*	10	< 1	6	2
Wetlands	5000 km^2^	1.19	8	3	5	2	*	3	18	5	1

* Not listed; ** Pathogens are listed as # 2 cause and habitat alterations #3 in lotic systems; *** Excluding Great Lakes.

### 2.2. Hydrologic Alteration and Habitat Destruction and Impairment

Major ecosystem changes accompany the conversion of land to agriculture [1]. Conversion of lowlands to agriculture typically begins with removal of trees and construction of ditches for water conveyance. Draining of wetlands for agriculture is repeatedly cited as the primary cause of wetland losses in a variety of different types of wetlands around the world [40,41,42,43,44,45]. There is a general consensus that the rate of wetland destruction in the 20^th^ century was unprecedented, especially in the United States. The area of bottomland hardwood forest in the Lower MAV, extending from extreme southern Illinois to the Gulf of Mexico, is currently less than one-fourth of its estimated area immediately following European colonization [46,47]. This decrease is almost entirely attributable to agricultural expansion [48]. 

Examining the entire contiguous United States, Dahl [41] estimated that half of the historical wetland area had been drained and primarily converted to farmland. These practices result in increased sediment loading to receiving waters [49], and decreased surface storage that can lead to downstream flooding [50]. Furthermore, these effects are compounded by flood control efforts related to stream leveeing and channelization [51,52]. Conversely, localized changes in hydrology resulting from channelization can cause desiccation of wetlands dependent upon overbank flooding [53]. Additionally, stream incision can lower local water tables, with a resulting loss of groundwater-fed wetlands and a decrease of wetland hydroperiod and inundation duration [54,55].

**Figure 2:**
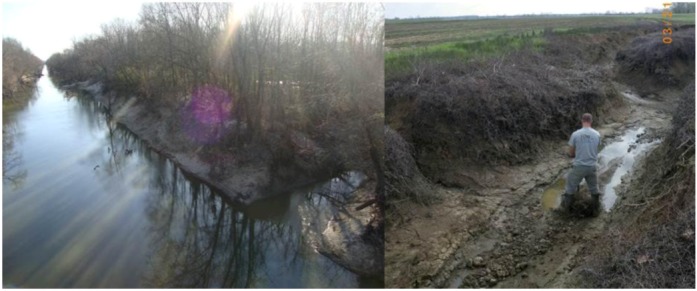
Agricultural drainage can cause stream incision at multiple spatial scales. At left, the Loosahatchie River, north of Memphis, Tennessee, USA. Channelization has resulted in stream incision, not only of the main channel, but also of tributaries, as seen by the difference between the water level and top bank of the stream flowing into the main channel. At right is an agricultural drainage ditch near Yazoo City, Mississippi, USA. Although the channel is sinuous, high peak flows have resulted in bed degradation and mass wasting. Although this photo was taken shortly after a substantial rainfall event the channel holds little water.

In low-lying areas amenable to extensive row-cropping, forests and perennial grasslands are replaced with annual crops, leaving the land unvegetated for much of the year. It is well established that removal of vegetation leads to erosion, particularly when followed by recurring conventional tillage [56,57,58,59,60,61]. Historically, even following changes in vegetative land cover, fluvial processes, along with establishment of aquatic or riparian vegetation, have somewhat ameliorated these impacts. Extensive channelization, however, has resulted in stream incision, which disconnects lotic systems from their floodplains, concentrating into narrow channels the energy that would be dissipated as sheetflow across riparian zones (Figure 2). The resulting elevated water velocity not only increases erosion, but also effectively prevents establishment of vegetation at the aquatic/terrestrial interface. Furthermore, because ditches lack the heterogeneity of riverine systems, there are few snags, point bars or pool-and-riffle complexes where sediment would typically accumulate in wider downstream reaches [62]. This lack of heterogeneity, in combination with channel instability and high levels of fine sediment, decreases diversity of fish [63,64,65,66] and mussels [66,67,68,69]. When combined with removal of perennial vegetation, areas under artificial drainage store less water, increasing flow variability and peak discharge [70,71,72]. This increased discharge leads to further channel incision, head cutting, and stream bank erosion via mass wasting [72,73,74]. Channelization of a given stream reach can thus lead to incision either upstream or downstream as a result of head cutting or increased peak discharge during storm events [75,76]. 

The cumulative geological and hydrological impacts of extensive drainage networks are difficult to ascertain with any level of precision, but are nevertheless substantial. Simon [77] documented relatively recent morphological changes in the Obion and Forked Deer Rivers in western Tennessee including a decrease in channel length of 44%, increased bed gradients of up to 600%, and lowering of channel beds by as much as five meters. These changes likely accounted for a substantial portion of the estimated 11 million cubic meters of sediment this system delivered to the Mississippi River in the 20 years preceding the study. The consequences of increased sediment transport, deposition, and suspension include increases in turbidity, respiratory and feeding impairment of aquatic fauna, and increased scouring of both macrophytes and the channel itself. Additionally, increased sediment load is associated with increases in phosphorus loading, as the principal form of mineralized phosphorus, orthophosphate, is primarily bound to fine sediments, as opposed to being dissolved in the water column.

In addition to channelization, other water management practices, such as subsurface drainage, change groundwater processes. Surface drainage is simply the practice of straightening or increasing the volume of existing channels, or creating ditches by removal of earth, and evidence for its implementation can be found among the earliest evidence of civilization. Subsurface drainage, although found in urban archeological sites, has only been applied in an extensive agricultural setting fairly recently. Historically, it involved placement in low areas of water-permeable clay tiles, which drained into subsurface pipes, which, in turn emptied into adjacent streams or ditches. More recently this approach has been replaced with subsurface perforated, corrugated plastic pipes, which essentially lower the vadose zone in a field, allowing enhanced root penetration for crops. 

Subsurface drainage has several benefits over surface drainage with regard to receiving water, including decreased introduction of suspended sediment and the phosphorus that is often associated with it [59,78,79]. However, likely due to decreased potential for interaction with surface vegetation and with the oxidation-reduction gradients and associated microbes that are common in saturated soils, subsurface drainage leads to higher concentrations of nitrate as well as dissolved phosphorus in agricultural runoff [79,80,81]. These differences depend to a large degree on the depth of the vadose zones and soil hydraulic conductivity, as well as management practices related to fertilizer application and tillage. As of 1987, it was estimated that 35% of artificially drained agricultural land in the United States utilized subsurface drainage [26]. This percentage has undoubtedly increased considering increases in crop productivity, particularly corn (*Zea mays*), in the past 25 years. Although a recent comprehensive survey is not available, estimates in the midwestern United States, where subsurface drainage is most intensive, estimated that as of 1998 an additional 70,000 hectares of subsurface drainage for agriculture was installed in the states of Ohio, Iowa, and Minnesota; whereas there was no net gain in the states of Illinois, Indiana, Michigan, or Missouri [82]. In contrast, a more recent estimate utilizing GIS analyses of soil drainage class suggested a significant increase in Illinois, whereas the areas of subsurface drainage Iowa and Ohio were similar to 1987 values [83]. Subsurface drainage is also a common practice in Europe [2,84]. Given the increased yield associate with subsurface drainage, it is likely to remain a fixture in landscapes of extensive agriculture, demanding that new approaches for nutrient interception are implemented.

Another cause of hydrologic alteration is irrigation, either from surface or subsurface sources. Although the ecological impacts resulting from overconsumption of surface water are readily apparent, the long-term impacts of excessive groundwater removal are no less dire, and likely more difficult to repair. Worldwide, irrigation accounts for about 70% of freshwater withdrawal [85]. Less than half of this water is estimated to come from groundwater sources, but in arid areas with no history of surface irrigation practices, such as Australia, nearly all irrigation water is from groundwater [85]. Use of groundwater for irrigation is a growing practice, and at current usage rates many aquifers are not being managed as a renewable resource. Given uncertainties about the effects of climate change on the need for irrigation and on aquifer recharge, these practices are a growing concern.

With regard to drainage practices, irrigation not only supplies ditches with runoff during dry periods, but can also desiccate streams by lowering the water table, decreasing base flows and resulting in decreased discharge and greater fluctuations in flow [54]. Although most of the research in this area has focused on streams in arid and semi-arid environments [85,86], the same trends have been observed in streams of mesic environments with porous substrata, for example karstic systems [87]. Factors related to stream hydrology are among the most important determinants of the resulting communities and can even impact riparian trees [88]. Additionally, changes to hydrology can subsequently affect channel morphology and other environmental parameters important to stream biota such as temperature and dissolved oxygen. It is uncertain how groundwater depletion would affect agricultural drainage ditches, but in systems with high surface-groundwater connectivity impacts are likely to occur. 

### 2.3 Nutrient Enrichment

In addition to land cover changes and hydrologic alterations, modern agriculture contributes to environmental degradation via nutrient enrichment. Anthropogenic nitrate fixation exceeds background fixation from natural sources [89], about 15% of which enters rivers as nitrate [90]. A recent review cites a number of studies in Europe that trace between approximately 35%–80% of riverine nitrogen load to agricultural inputs [91]. Nitrogen loading to the Gulf of Mexico via the Mississippi River doubled over the second half of the 20^th^ century [92]. Increases in riverine nutrient loads are not only caused by increased fertilizer application rates across the agricultural landscape, but also by the hydrologic alterations of channelization that lead to increased sediment and dissolved nutrient loads. Due to increased discharge rates and decreased biogeochemical processing, narrow, incised channels tend toward higher concentrations of both dissolved and particulate-bound nutrients, such as orthophosphate, than streams that retain some hydrologic connection to their historic floodplain [93]. Phosphorus loading rates to coastal waters toward the end of the 20^th^ century were calculated to be about three times the estimated historic background rates [94]. Furthermore, as previously mentioned, agriculture affects subsurface hydrologic processes, which can impact chemical speciation, making drainage ditches a conduit for bioavailable dissolved nitrate and other soluble pollutants [95].

Nitrate concentrations of 10 mg/L (the current maximum for drinking water allowed by the US government) have been demonstrated to be detrimental to invertebrates, fish, and amphibians [96]. Reduced species of nitrogen, such as ammonia and nitrite, can be far more toxic. A large body of research has demonstrated toxic effects in fish [97]. Although ammonia is the predominate form of nitrogenous waste produced by fish, fish may actually be more susceptible to ammonia toxicity than invertebrates [98]. Ammonia also causes decreased fitness in amphibians [99,100,101]. In general, direct amphibian mortality occurs at concentrations that would be considered high even in an agricultural setting [100]. Not surprisingly, however, toxicity varies among species. Green frog tadpoles (*Rana clamitans*) exposed concentrations of nitrate 5 mg/L–20 mg/L demonstrated a significant increase in mortality compared to those reared at lower concentrations [101]. Even aquatic plants, such as rice (*Oryza sativa*) which would be expected to thrive under a high-nitrogen regime, can display toxicity under high concentrations of ionic ammonium [102]. The distinction between ammonia and ammonium is important, as ammonia is toxic at lower concentrations. The more toxic form, ammonia, predominates in water with higher pH, leading to the development of pH-dependent toxicity criteria [103].

The greatest impact of nutrient enrichment to aquatic ecosystems is not the direct toxicity of fertilizers, but the complex set of environmental responses referred to as eutrophication [104,105]. An understanding of the process of eutrophication relies upon first an explanation of the concept of limiting nutrients. In any ecosystem, organisms must concentrate some elements at proportions far higher than their environmental availability. For primary producers, these elements are often in the form of mineral nutrients; and, because the need for nitrogen and phosphorus is relatively high when compared to their bioavailability, they are the nutrients that most often limit the growth of autotrophs. In coastal or marine systems, nitrogen availability usually limits production. In freshwater systems, phosphorus generally limits production. While these generalizations certainly do not apply to all times and places, they do occur commonly enough for them to serve as the basis for understanding how excess nitrogen and phosphorus impact both intra-continental waters and the coastal waters they sustain.

Although coastal systems have received the widest audience with regards to impacts from agriculture, inland waters are subjected to the most direct impacts, not only in terms of habitat loss and hydrologic alterations as previously discussed, but also with regard to nutrient enrichment. In general, nutrient enrichment has been considered a greater problem in lentic systems, such as wetlands and lakes, than in flowing waters [104]. However, decreased macroinvertebrate biodiversity does result from stream nutrient enrichment, caused either by nutrient toxicity, or depressed oxygen levels resulting from the increased respiratory demands of eutrophic systems [106]. For example, poor water quality, along with habitat alteration is among the most commonly cited causes of mussel declines [69]. 

In aquatic systems, increased sediment load increases turbidity, thus decreasing light penetration into the water column in two ways. First, the suspended particles can directly intercept light near the surface. Second, even long after settling occurs, sediment can be a source of phosphorus enrichment due to dissociation during periods of anoxia, a process referred to as “internal loading” [107], leading to repeated cycles of phytoplankton blooms that are essentially indefinite. In systems that are historically mesotrophic or oligotrophic, the increased productivity near the surface of the water compresses the photic zone toward the surface, essentially extinguishing deep water submergent plants and benthic algae, as well as depressing recruitment of new littoral emergents. As these plants comprise a key structural component in the water column of many aquatic environments, their loss has a profound effect on the resulting invertebrate assemblages [108]. The majority of the research examining these eutrophication-related effects was conducted in lakes, but similar changes have been observed in experimental ditches with a static water level [109]. Even in the case of wetlands dominated by dense stands of emergent plants, changes in vegetation can result from differing competitive ability at higher nutrient concentrations. For example, along with hydrologic changes, eutrophication is a major factor in the current shift from sawgrass (*Cladium jamaicense*) dominated marshes to cattail (*Juncus domingensis*) dominated marshes in the Florida Everglades [110,111]. 

Because eutrophication decreases the depth at which photosynthesis can occur, and increases ecosystem respiratory demands, it tends to cause periods of low oxygen availability, or hypoxia. Diaz [112] defines hypoxia as dissolved oxygen concentrations equal to or less than 2 ppm, as at these levels of dissolved oxygen only specialized organisms can thrive. In other words, at these levels, oxygen often becomes the factor that limits biodiversity, if not productivity, of an aquatic system. In the photic zone of the water column, photosynthetic creation of oxygen predominates during the day, while respiratory oxygen uptake predominates at night. At increasing depths, photosynthetic oxygen production declines, a trend that is amplified as eutrophic conditions increase phytoplankton densities, thus leading to greater light absorption near the surface. Eutrophication not only compresses the photic zone, causing a decrease in the potential for deep-water oxygen production, but by increasing organic carbon deposition to the benthos it causes a corresponding increase of oxygen use by benthic decomposers. This combination of increased respiratory demand and decreased oxygen production results in hypoxic conditions that are seasonal, rather than diel. Although seasonal oxygen depletion is a natural feature common to continental shelf ecosystems, temperate lakes, and wetlands, anthropogenic eutrophication has resulted in a significant increase in the size, duration, and magnitude of these events [113].

In aquaculture, it is a common practice to add nutrient amendments to increase yield. Similarly, research has demonstrated a linkage between nitrogen loading into coastal systems and total yield of their fisheries, particularly with regard to pelagic fish [114]. The strength of this relationship, however, has been called into question, as the increases in primary productivity resulting from nutrient enrichment have not been demonstrated to increase the biomass of higher trophic levels [115]. More recent research suggests that much of the increase in productivity actually yields biomass that is inedible or unavailable to primary consumers, and is cycled through microbial processes, rather than contributing to production in higher trophic levels [116]. One striking example is the explosive blooms of toxic dinoflagellates in response to eutrophication, leading to the well-known “red tides” that have long been associated with mass fish kills [117]. Both coastal and continental systems may be affected by many forms of toxic phytoplankton, including cyanobacteria, some of which can actually exacerbate eutrophication via fixation of atmospheric nitrogen [117]. 

Eutrophication is one of the primary causes of degradation of coastal ecosystems [118,119], and agriculture is the primary culprit implicated in hypoxic zone development in a wide array of coastal ecosystems, including the northern Gulf of Mexico, the Northern Adriatic Sea, the Kattegat, the Baltic Sea, and the northwestern continental shelf of the Black Sea, among others [112,113]. In the Kattegat, between Sweden and Denmark, the result has been a precipitous decline in stocks of the Norway lobster and Atlantic cod [120]. In the both the Baltic Sea and the Black Sea, the impacts on fisheries have been profound, leading to nearly a 75% decrease in the species available for commercial fisheries on the northwestern continental shelf of the Black Sea and a general ecosystem-level change in the Baltic Sea fisheries [121,122] . Although a general increase in the extent of the hypoxic zone in the Gulf of Mexico has been recorded [4], there has been less consensus on the resulting effects on fisheries [112]. A number of recent studies have documented changes in fisheries in the northern Gulf of Mexico, including a shift in predominance from demersal to pelagic fisheries, which may have economic impacts on brown shrimp (*Farfantepenaeus axtecus*) and Atlantic croaker (*Micropagonias undulatus*) fisheries [123,124,125].

## 3. Ecological Restoration

As more evidence of the impacts of agricultural drainage is documented there is growing concern among the general public, prompting government involvement in the management of privately owned lands. Agricultural management practices that focus on conservation are becoming widely accepted, including long-term easements that remove marginal land from production. Specific practices for water quality improvement are being implemented and researched in North America, Australia and Europe. A variety of government conservation programs involve practices meant to address impacts at multiple spatial scales, but are generally motivated from the perspective of receiving waters, rather than local impacts. The Gulf of Mexico Action Plan [126], for example, called for a reduction of the Gulf of Mexico hypoxic zone to a mean extent of 5,000 km^2^ by 2015 [127]. To implement this target, the action plan and task force recommended in 2001, that a 30% reduction of total nitrogen was needed. A revision to the 2001 action plan [128], based on model predictions has elevated nutrient loading reduction targets for both nitrogen and phosphorus to 45%. Decreasing nutrient loads and concentrations leaving farms not only has effects on coastal ecosystems, but also on continental aquatic systems including lakes, rivers, and wetlands.

### 3.1. Wetland Restoration

Because a large portion of current lowland agriculture is located on former wetlands, one approach toward improving water quality and increasing biodiversity is reconversion or restoration of marginal agricultural land back to historical wetland types. This approach is attractive, as a large body of evidence exists demonstrating the importance of wetlands for wildlife, flood control, and remediation of non-point source pollution, particularly with regard to nitrogen removal [129,130,131]. Brinson and Eckles [14] recently summarized one of the first broad-scale reviews of federally supported wetland restoration programs in the United States, observing that, from a qualitative perspective these programs have had a positive environmental impact, but the degree of these impacts varies by region and by program. 

Wetland restoration of agricultural land is most likely to occur in areas of marginal productivity where even extensive drainage efforts have been ineffective. However, the current rate of restoration cannot continue without expanding into high-production agricultural land in the coming decades. Additionally, restoration efforts too often suffer from flaws in assumptions and applications [132]. Thus the success of wetland restoration has been inconsistent, and even after decades of naturalization, restored wetlands generally do not compare favorably with reference wetlands in terms of biogeochemistry or biodiversity [133]. This disparity is especially strong in areas where the landscape has undergone extensive hydrological alterations that disconnect streams from the surrounding landscape, which may preclude restoration attempts to recreate historical conditions [14,134,135]. 

**Figure 3:**
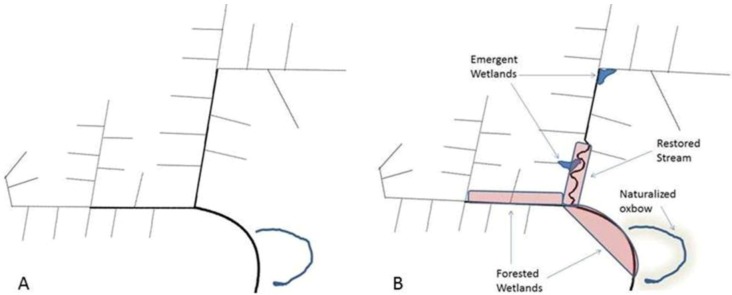
**(a)** Schematic of a standard agricultural drainage system representing approximately one hectare. Primary streams (*i.e.* – edge of field ditches) are cropped to the edge or bordered by grass buffer strips. Higher order streams may have riparian trees, depending upon climate and agricultural management. The system is designed solely to convey water. **(b)** The same drainage following extensive restoration. Wetland and stream restoration in systems impacted by agriculture strategically put effort into restoring wetlands in areas that are marginal for agriculture and into increasing sinuosity and floodplain width in second and third order streams. Management practices range from simply ceasing agriculture in flood-prone areas to planting and promoting desired plant species using prescribed floods and drawdowns. Extensive earthmoving is often a prerequisite if the streams are disconnected from their floodplains or for the intensive water level management recommended for wildlife habitat. Often, even highly engineered restoration projects meet with only moderate success due to infrequent hydrologic connectivity or geomorphic instability caused by high discharge events. Although a large area is incorporated into the restoration plan, several kilometers of stream reach is upstream of the restoration influences.

In those low-lying areas that do intercept agricultural runoff, any restored wetlands would be subjected to the same non-point source pollutants that contribute to wetland functional impairment. Finally, the creation of large wetlands in predominantly agricultural landscapes can have unforeseen outcomes with respect to wildlife-mediated impacts on nutrient cycling. Benthic fish, for example, can resuspend phosphorus-laden sediments in shallow lentic systems, leading to increased internal phosphorus loading [136]. Geese, which congregate in the tens of thousands in wetland-agriculture mosaics, can mobilize terrestrial nutrients, thus increasing aquatic loading rates, or directly deposit fecal material into wetlands [137,138]. With respect to these many difficulties associated with agricultural wetlands, we contend that their function as biodiversity hotspots and “kidneys of the landscape” will be limited unless they are integrated into a comprehensive plan including conservation agriculture and the management of artificial or channelized low-order agricultural streams (*i.e.*, drainage ditches (Figure 3)).

### 3.2. Riparian Buffers

A number of conservation-oriented land management practices, such as cover cropping and conservation tillage, have been implemented in areas of extensive agriculture and have been demonstrated to improve local water quality [139]. Other practices, including frequency of crop rotations [140], organic farming [141], and pesticide application [142] have a wide range of effects on the ecosystem functioning of agricultural headwaters, including ditches. The overall impact of many popular and generally effective practices, however, can be relatively less important on habitat quality and non-point source pollution transport than in-channel conditions [139,143,144]. Because the area of land immediately adjacent to a stream or ditch, the riparian zone, has such a great influence over stream functioning though, its management merits discussion. 

Riparian buffer strips can be an effective method against some types of nonpoint source pollution entering aquatic systems, especially along low-order agricultural streams in areas with little topographic relief, and buffers can also be important as corridors for terrestrial animals [145,146,147]. Although grass buffer strips as narrow as five to ten meters are effective for removal of sediment-bound nutrients in surface flow effluent [148,149,150], when used in isolation of other conservation practices their overall impact on water quality may be limited [144]. Because herbaceous buffer strips result in localized sedimentation, they tend to accrete sediment over time, resulting in berms that concentrate the flow path of runoff into shallow, naturally forming channels [151]. This issue has led to modifications of buffer designs, including substantively different approaches such as grass swales described below in Section 4 *Instream Drainage Management*. 

Such modifications include not only changes in hydrologic design, but also ecological restoration approaches that increase the diversity and structural complexity of these vegetated buffers by creating multiple zones of vegetation [6,146,152,153,154] . The general consensus is that zoned buffers are more effective buffers, because different types of vegetation have different mechanisms for improving water quality. Riparian hedgerows, for example, by excluding livestock from streams, can improve water quality [155]. Given the large volume of research demonstrating the impact of riparian trees on nutrient cycling and loading into streams, tree buffers are likely to have long-term benefits for water quality and habitat in many agricultural streams. Trees can draw nutrients from deep below the soil surface unavailable to many mesic forbs and grasses, thus sequestering nutrients from subsurface flows and enhancing denitrification of soil water and stream sediments [57,156,157,158]. Although such studies are often cited as justification for using trees in agricultural buffers, others have found no differences among vegetation types [159] or greater nitrogen retention in grass buffers [160]. 

Buffers including trees are advocated both for acting as corridors linking terrestrial habitats, as well as themselves serving as habitat [147]. Likewise, riparian afforestation of agricultural headwaters is often recommended for aquatic habitat improvement of degraded agricultural streams; however, recent research has suggested that for long-term enhancement of aquatic habitat, resources would be better utilized by investing in improving riparian areas in existing downstream floodplains [16]. In addition to providing structure and carbon inputs, during the growing season deciduous trees shade the water, lowering temperatures and decreasing nuisance macrophytes [161]. Streams with forested riparian zones often have greater productivity and diversity of aquatic macroinvertebrates and understory plants [158,162,163]. These same properties, however, can greatly impact stream morphology and ecological functioning. Studies finding improved habitat and nutrient cycling associated with forested riparian zones should be viewed with two caveats. The first caveat is that these studies are usually carried out in areas with a well-developed canopy and understory, which can take several years to develop. The second caveat is that some of the instream benefits associated with closed canopy streams are measured over the length of the stream, rather than the area of the stream [158]. The first point is important with regard to creation of buffer zones, in that any improvements in habitat or nutrient reductions provided by planting trees may take several years to develop. The second point is important because forested streams are generally shallower and wider than their counterparts in open fields [154,158]. The implication is that some degree of the differences observed between these two types of system result indirectly from the effects of vegetation on stream morphology, as opposed pathways related more directly to nutrient cycling.

### 3.3. Limitations to Restoration of Agricultural Headwaters

The impacts of agricultural drainage practices are extensive. As such, proposed mitigation responses involve addressing impacts hierarchically, integrating spatial scales ranging from a single field to major river basins that drain large portions of entire continents [10,12]. However, lowland headwaters merit special attention with regard for their potential for remediation for a number of reasons. Perhaps the most straightforward reasons are that headwaters comprise the greatest proportion of stream reach [164] and have been most severely altered. In the midwestern United States, for example, headwaters of the Mississippi River System that were formerly fed by small ephemeral wetlands are now fed primarily by artificial drainage systems that utilize subsurface piping [2]. Farther south, in western Tennessee and northwestern Mississippi, practically all of the lowland headwaters are artificial ditches or completely channelized streams [165]. Small-scale drainage projects were more readily capitalized upon by individual landowners and small consortiums than were extensive flood-control efforts, due to the general tractability of smaller projects, both in terms of construction and politics. It is reasonable to assume that small-scale restoration projects in headwater streams are likewise more tractable, especially with regard to determining their effectiveness through replicated experimental studies, as described by Smiley *et al.* [166]. In larger streams and rivers such replication is impractical or impossible [139,167]. Additionally, although pollutants in agricultural runoff are considered “non-point source,” the general locality of inputs can be determined, allowing more precision, and thus efficiency, in placement of mitigation practices [14,168]. 

There are other reasons related to the inherent physical and ecological processes of aquatic systems that make lower order systems an appealing focus for mitigating pollutants. Because of their higher surface area : volume ratios and higher retention time relative to total discharge, smaller streams and ditches would be expected to have a relatively greater capacity for biogeochemical processing of nutrients than high order streams [17]. This supposition is supported by research demonstrating that nitrogen losses in the Mississippi River system are inversely related to channel size, indicating increased capacity for nitrogen removal in smaller streams [169]. Under current drainage practices, a large degree of this capacity for nutrient remediation may be unrealized due to design and management focused solely on water conveyance. Because net pollutant flow in lotic systems is unidirectional, practices focusing on higher order streams essentially ignore the potential habitat value of upstream tributaries. Additionally, in higher order streams, anthropogenic factors originating upstream can be more important than local conditions in determining the health of riverine habitats and water quality [170]. 

When examining a drainage basin as a continuum from receiving water bodies to headwaters, in agricultural systems there is inevitably a point at which the system is not simply altered, but fundamentally different from its historical state in terms of hydrology, morphology, and ecology. Hydrologic alterations and related changes in channel morphology include the trend toward increased channelization of flow from systems that were historically sheetflow wetlands or highly sinuous streams. The concept of a transition from allochthonous carbon inputs to autochthonous carbon production as wider streams become less influenced by riparian canopy, which was codified as the “river continuum concept” by Vannote *et al.* [171], does not apply to systems fed by artificial drainages subset in, or at the edge of agricultural fields. Even in former prairie land where canopy played a less important role in the biogeochemistry and ecology of headwaters, drainage impacts on hydrology mean that former spring-fed streams are now fed by the aforementioned subsurface drainage pipes. Because the character of a stream is directly influenced by the adjacent landscape, and because trends in restoration are moving toward more naturalistic approaches, we will revisit the topic of woody riparian buffers. 

From the standpoint of stream restoration, riparian afforestation does not accurately reproduce historic conditions of many headwater stream reaches in agricultural areas, such as large portions of the Interior Plains of North America [149]. Such plantings can interfere with the functioning of subsurface drainage, which they are unlikely to effectively intercept [146]. Arango and Tank [172] found that riparian buffers had no effect on stream nitrate concentration during the growing season in areas under subsurface drainage. Even in areas such as the southeastern United States, where the diversity of headwater fisheries are highly dependent upon the input of woody debris [173], there are potential costs that in many cases may outweigh the benefits of riparian trees. 

One potential problem of riparian reforestation within an agricultural matrix relates to the issue of stream incision *versus* steam widening. Open, low-order streams with high light infiltration tend to have comparatively stable banks as a result of vegetative ground cover [149]. Although tree cover has been correlated with increased understory diversity in buffers [162], the vegetation would likely be sparser and clumped. Over several years the result is that gullies can develop along the top bank, not only negating buffer effectiveness, but also enhancing bank erosion, widening the stream and infilling the main channel [149,154] (see [6] for opposing view). Because such streams tend to flood regularly, there are practical concerns related to their impact on agricultural productivity. A more immediate issue is the depression of macrophyte productivity [6,161,174,175]. The short-term effect of macrophyte shading on nutrients varies depending upon location and the nutrients of interest [174,175,176]; however in systems with soft substratum, the resulting decreases in macrophyte cover may initially be associated with higher rates of in-channel erosion [177]. 

Other potential costs of tree buffers are related to agricultural management. Buffers including trees are wider than other buffers, withdraw more water, and may shade field margins. The input of woody debris into streams is a good thing from an ecological viewpoint, but the resulting flow constrictions may be difficult to remove if trees or shrubs block stream access for machinery. The height of buffers within fields may be limited by center-pivot irrigation, a growing practice throughout the United States of America. Tree buffers are recommended for decreasing by-spray into aquatic environments [178], but adoption of this practice may be limited by the prevalence of aerial herbicide application in some areas, due to herbicides affecting tree establishment, or the presence of standing trees limiting accessibility to crop dusters. Although such considerations are rarely taken into account in scientific articles promoting riparian forestation, they are almost certainly important for those responsible for managing farms. A survey of farmers in Michigan, USA, found that they significantly preferred grass buffers over hedge or woody buffers, and nearly twice as many actually implemented grass buffers as tree buffers [179].

Other limitations are related to how the landscape impacts channel morphology. Attempts at rehabilitating ecological functions of drainage ditches must consider three related traits linked to channel morphology: gradient, substratum, and hydrology. Gradient and substratum are less subject to small-scale manipulation than hydrology, though no less important with regard to their impact on outcomes. In areas of essentially zero gradient, hydrology may be less dynamic than in low-relief ridge and swale landscapes. In areas like the Netherlands or Florida, ditches generally have consistent flow year round [180,181]. In contrast, in areas with even slight elevation gradients, such as the “Mississippi Delta” region of the United States, ditches experience drastic changes in hydrology, both seasonally and in response to precipitation events. Although channelization has been implicated as a factor in increased hydrologic variability, both comparative studies and conceptual models have also demonstrated the opposite trend [76,182,183]. There are a number of potential factors that may explain why channels that have been altered to convey water have more persistent flow compared to unchannelized streams or stream reaches. 

Channelization of rural headwaters is usually limited to flatter landscapes, indicating that some differences in flow persistence may primarily result from higher stream gradients or greater watershed relief in unchannelized streams (Figure 1). Often riparian tree removal proceeds channelization. Because trees have deeper roots and higher canopy transpiration rates than other riparian plants, they can lower subsurface water tables [184], subsequently decreasing stream flow [185]. Perhaps the most likely explanation in areas with a high water table is that when the channel is entrenched it intercepts the more stable groundwater reserves [183]. This connection between surface and groundwater, referred to as hyporheic exchange, is an important, and often overlooked aspect of the chemistry and ecology of surface waters.

Restoration of hydrologic conditions prevalent in unchannelized headwater streams is not a likely outcome for ditches draining land in agricultural production for two related reasons. First, unchannelized headwater streams are characterized by frequent overbank flooding in response to precipitation [76,182], which would have obvious implications on crop yield. Second, in comparison to channelized streams, unchannelized headwater streams in lowlands are also characterized by comparatively greater sinuosity resulting from stream meandering across the floodplain. It is unclear how such natural sinuosity could be reestablished within a reasonable timeframe, especially for incised streams wherein the historic floodplain has been converted to agricultural production. In any case, such efforts would require significant monetary investment and would remove large amounts of land from agricultural production

## 4. Instream Drainage Management

Generally, instream nutrient removal on non-point source pollutants is low, especially for dissolved nutrients, such as nitrate. Reviews on nitrate removal estimate about 5% losses due to denitrification in any given reach of an agricultural headwater stream [13,91]. Although these rates usually increase with increasing nitrate concentration, such increases plateau well below concentrations typical for drainage ditches [13,186,187]. This plateau effect has led some researchers to view riparian buffers as the last bastion against enrichment from nitrogen fertilizers [188].

When viewed from a the perspective of potential removal of multiple types of non-point source pollution from agriculture, however, instream approaches to decreasing downstream impacts, show a great deal of promise. Consider, for example, that the majority of suspended sediment in many lowland agricultural headwaters originates from channel and bank erosion [139], and that instream practices intercept non-particulate bound nutrients whether they are conveyed by subsurface drainage systems or surface runoff. 

In light of widespread acceptance that primary and secondary agricultural drainage systems show potential for mitigating the very impacts they cause, a new emphasis on alternative drainage management and design is arising for remediating local and downstream impacts. Two approaches that intercept the concentrated flow paths of water and that have been demonstrated to decrease non-point source pollution or provide aquatic habitat are swales vegetated with either upland or wetland grasses [189] and drop pipes in eroded gullies [190,191,192]. Off-season flooding of agricultural fields via controlled drainage using flashboard risers has long been used to attract waterfowl, and may stabilize hydrology and improve water quality in headwaters, at least during times when the land is not in agricultural production [193]. A similar structure used passively that intercepts runoff in concentrated flow paths is a slotted pipe, simple a standard drainpipe with the bottom of the inflow blocked to stop bedflow of unconsolidated sediment. 

**Figure 4:**
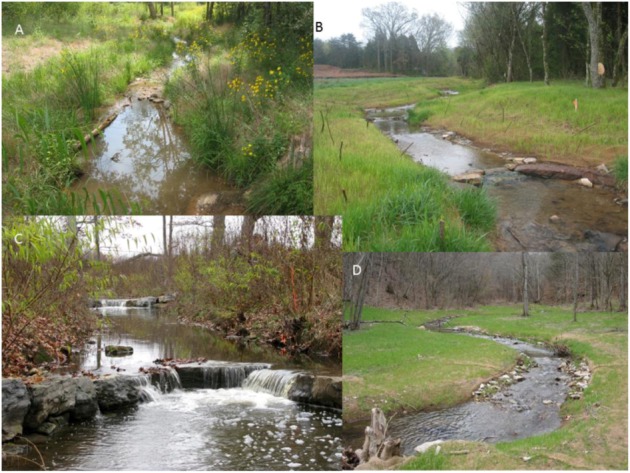
Structures used for stream restoration. **A**. A small log vane is used to stabilize the bank immediately upstream of a small constructed riffle. **B**. Cross-vanes are used for bed stabilization. Newly planted willow stakes line the bank and constructed mini-floodplain. **C**. Cross-vanes and established willow stakes. **D**. Root-wads, constructed riffles, and artificial meanders.

Similar practices are being developed for incised ditches that experience more regular patterns of inundation and flow. There are a variety of specific instream practices used for stream restoration that may be applied to or modified for use in drainage ditches (Figure 4). Such practices can be broadly categorized by two basic approaches: creating small instream wetlands or pools using flow impediment structures or altering flow to “naturalize” the streams via miniature inset flood plains. While both approaches seek to increase hydrologic and geomorphic stability of the system, they differ in that stream-type systems rely upon regular discharge creating fluvial processes for “self-cleaning” and re-deposition of sediment, while wetland-type systems rely upon periods of low discharge for the settling of sediment as a result of decreased water velocity. Both approaches rely upon the establishment of wetland or aquatic macrophytes as integral components to increase system stability and decrease non-point source pollution. 

### 4.1. Two-stage Ditches

Hydrology is probably the most important factor in determining both nutrient transport and macrophyte composition, and should be the determining factor in whether a fluvial or wetland approach is used in a given reach. In ditches with extensive hyporheic inputs or perennial flows, a design stemming from Rosgen stream restoration methods [194], such as a two-stage design ditch, is one alternative. Proponents of this design offer it as a practical compromise that imparts some of the physical characteristics of streams to drainage ditches without compromising their function as water conveyances [195,196]. These systems are comprised of a small low-flow channel inset within a wide-profile flood channel. The inset channel forms natural meanders, which balance processes of erosion and sedimentation; whereas the large channel effectively conveys floodwaters without leading to bank sloughing. Additionally, during periods of high water when the majority of nutrient transport occurs [187,197,198], the “instream benches” that are essentially a miniature vegetated floodplain, would be expected to improve water quality in the same way as buffer strips or grass swales. Because the effectiveness of buffers is limited by their degree of connectivity to the aquatic environment [153], these vegetated benches, essentially acting as saturated buffers would actually be expected to function more efficiently than many traditional buffers.

Although still a matter of some debate, there is documentation that supports improved nutrient reductions in two-stage ditches, including peer-reviewed research [199,200,201]. Over time, fluvial processes tend to stabilize channel integrity [77], and channelized ditches and streams can naturally develop two-stage morphology [196,202]. Sediment collected from naturally-formed benches exhibited twice the denitrification rates of sediment from the slopes of standard trapezoidal ditches whether incubated under ambient nitrate concentrations or concentrations of 100 mg/L [199]. This enhanced rate of denitrification likely results from higher organic matter in benches, as organic amendments increased denitrification in sediments from slopes of trapezoidal ditches, but not two-stage benches. 

Higher denitrification rates were also reported on benches of recently constructed two-stage ditches, which resulted in a 6%–9% decrease in nitrate export during storm flows [200]. One important aspect of this study is that during the year of higher precipitation instream denitrification was lower, whereas bench denitrification was almost double during the wetter year (6.1 mg N m^−2^ h^−1^ *vs.* 3.1 mg N m^−2^ h^−1^). These values fall in the 50^th^ percentile when compared with ranges estimated for surface-flow constructed wetlands [131] and exceeded instream rates, removing a conservatively estimated 89 kg N yr^−1^ over a 0.6 km reach. During periods of inundation, rates can be substantially higher, as the top 10 cm of bench sediment removed between 1.94 kg N d^−1^ –3.98 kg N d^−1^ over a one kilometer reach in contrast to 1.05 kg N d^−1^ removed by the top 20 cm of channel sediment [201]. 

Other research not yet subjected to extensive peer review reports even higher values. D’Ambrosio *et al.* [203] found nitrate removal rates on benches to be from 2–14 times the rates of channel removal, with rates exceeding 6 kg N d^−1^km^1^. Whereas over the course of 10 days, Kramer [204] observed a 14.3% decrease in nitrate load in a 1.9 km reach of a recently constructed two-stage ditch receiving about 750 kg N d^−1^, although limited data on the influence of tributaries implies the potential for a high degree of error. The basic two-stage design could be further modified for improved subsurface drainage interception by including miniature versions of riparian wetlands, such as the “horseshoe wetlands” described by Vought and Lacoursiére [84]. The presence of wetland plants during inundation of benches in two-stage ditches led to increased rates of denitrification [201]. 

Because of low aquatic biodiversity in traditionally designed ditches, the two-stage design has been advocated for improving habitat. The actual effectiveness of the two-stage design for local habitat restoration is still only hypothetical. A study of 33 sites in agricultural headwater streams found differences in community composition related to instream benches, but no relationship to measures of biotic integrity of invertebrate or fish assemblages [205]. It is important to note that there was also no difference found between agricultural drainages and reference streams. Although the overall body of research on two-stage ditches is limited, currently there are no known substantiated detrimental effects with regard to hydrology, biogeochemistry or ecology. 

Low-flow velocities can be higher in the somewhat constricted inset channels when compared to wider trapezoidal ditches. The decreased retention time and flushing of fine particulates may result in higher nutrient and sediment export during periods of base flow. Conversely, it has been suggested that the increased stability of two-stage ditches would likely decrease phosphorus transport during high flows [206], but research is lacking. Although the two-stage design potentially removes a small amount of land from production by widening drainage pathways, the area is comparable to or less than that recommended for buffer strips. The main drawback to this approach is initial cost, as it requires some degree of engineering expertise and a great deal of precision earth-work. Increased stability, however, has been purported to make the two-stage design a better long-term investment than standard configurations [196]. Another potential problem is structural failure due to poor vegetation establishment or extreme peak discharge. Generally these systems have been applied in areas with subsurface drainage, which somewhat dampens storm peak discharge. In areas of extensive surface drainage, the intermittent or ephemeral ditches that feed two-stage systems may require further modifications that decrease velocity and sediment load, especially during vegetation establishment.

These modifications are essential upstream of deeply incised streams, because rehabilitation of incised streams is often set back by hydrology-dominated geomorphic processes. For instance in a 2^nd^ order stream rehabilitation study by Shields *et al.* [207], establishment of vegetation and embankment structures were ineffective for long term channel stabilization, due to high in-channel discharge. Although this failure may have been due in part to sharp elevation gradients between the primary and secondary streams in the study, the same processes may apply to low-gradient agricultural land. Extensive agricultural drainage, especially surface drainage, can lead to high peak discharge rates in receiving waters [54]. In areas of with a high density of ditches, increasing surface water storage via controlled drainage structures could have beneficial impacts downstream, by decreasing peak discharge. Additionally, these structures can be strategically placed around knick-points to decrease head-cutting and sediment loading.

### 4.2. Controlled Drainage

**Figure 5:**
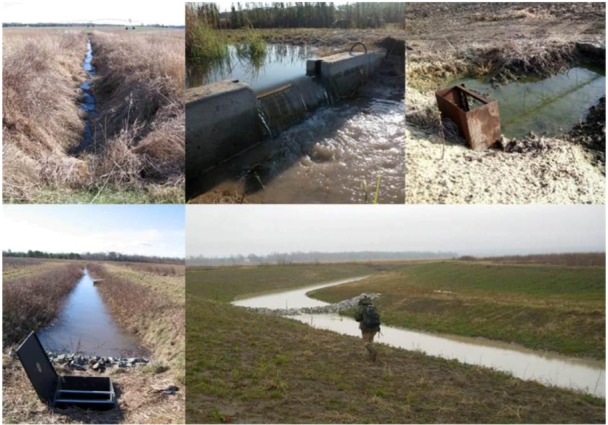
Various water control structures. *Far left*, downstream (top) and upstream (bottom) of a weir integrated into a ditch crossing on a farm on the Delmarva Peninsula, Maryland, USA (photos courtesy Dr. Tom Fisher). *Top center*, prefabricated slotted concrete weir in an experimental ditch system located on the campus of Arkansas State University. *Top right*, Flashboard riser with pipe draining into intermittent stream. Like the slotted concrete weir, boards can be added or removed to adjust the level of water retention. Although this structure is technically not located in a stream, it functions similarly in that it intercepts water in a channelized flow-path as opposed to sheetflow. *Bottom right*, demonstration ditch located near Yazoo City, Mississippi, USA. The terraces on the far bank are representative of a modified two-stage design, with the lower terrace being inundated during floods. This picture was taken during the rainy season. A smaller inset channel is not visible due to water depth. During the dry season the ditch is a series of shallow pools due to the presence of low-grade weir constructed of an earthen berms armored with rip-rap.

As opposed to approaches emulating lowland riverine systems, controlled drainage management is a management strategy whereby hydrological residence time is increased within the drainage path via impedance structures [208,209]. Originally “controlled drainage” was used to describe structures that intercepted subsurface drains such as riser pipes or flashboard risers [208]. The same or similar drainage control structures have also been implemented for surface flows (Figure 5, [198]). They can be situated at confluences in the flow-path, as slotted, drop or riser pipes or can be spatially gradated along the length of the ditch at intervals based upon slope and discharge as are grade-control structures [210]. All these structures work on the principle that increased hydrological residence and decreased peak flow velocities resulting from either impeding flow or capturing water volume will increase contact time of the water column with sediment substrate and vegetation [209]. This increased contact time improves opportunities for plant nutrient assimilation, microbial degradation of pesticides, hyperaccumulation of heavy metals and biogeochemical adsorption and precipitation of compounds with the sediment [211,212,213]. The actual removal rates for nutrients vary greatly among studies (Table 2). Other research has focused on using filters or mineral additives to immobilize specific nutrients [214,215]. Logistically and economically, there are a number of potential complications that may limit widespread adoption of these approaches; however, in systems with exceedingly high nutrient concentrations, such as those draining concentrated animal feeding operations, other methods may prove insufficient. 

Another important consideration is the effect of increasing structural complexity on surface water infiltration into the substratum. Areas of exchange between surface and subsurface waters, referred to as hyporheic zones, exhibit strong physical and chemical gradients. These gradients create biogeochemical “hotspots” for nutrient processing. In naturally meandering streams, horizontal hyporheic flows can occur in via downwelling porous substrata at bars and riffles [216,217]. When a stream reach is straightened, entrenched, and cleared of debris, these horizontal flows can be lost [217,218,219]. The result is that hyporheic processes depend primarily upon the hydraulic connectivity of the stream to groundwater, which is highly dependent upon substratum permeability [220,221]. Structures placed in the channel to impede surface flow for stream restoration can increase hyporheic exchange [222,223,224]. This increased exchange may or may not lead to elevated nitrogen in groundwater [225,226], but does tend to increase biogeochemical processing and alter surface water chemistry [227,228,229]. Impacts on nutrient reductions, specifically nitrate, are uncertain and likely depend upon organic carbon availability in the substrate as well as hydraulic conductivity [227]. Using impediment structures promote subsurface flows through organic materials have been demonstrated as a highly effective method for nitrate removal in drainage ditches [230].

**Table 2:** Decreases in nutrient loads using drainage management structures.

Location and basin or plot size	System type	Structure	*Nutrient Elemental Concentration (mg/L)		*Load (kg ha^−1^ yr^−1^)	**% load Decrease	Reference	Duration
Ontario, Canada; 3.5 ha basin	corn/soybean subsurface drainage	Riser pipes at 25 & 50 cm above free flow	Nitrate	>8–16	>0.8	62%–95%	[231]	2 years
North Carolina, USA; each plot 3–16 ha	Corn subsurface drainage	Flashboard riser ~30–50 cm from soil surface	Nitrate	>2–17 (upper range)	>25–40	50%–85%	[232]	3 years
North Carolina, USA; multiple studies	surface drainage	Controlled drainage	TN		>14 kg/ha^−1^	45%**	[233,234]	
			TP		>.05 kg/ha^−1^	42%**		
	subsurface drainage	Controlled drainage	TN		>31 kg/ha^−1^	44%**		
			TP		>0.2 kg/ha^−1^	20%**		
Chesapeake Bay, USA.; 80–90 ha basin	1’-2’ lowland suburban streams	Step-pools and riffles	TN	0.6–2.5	at low flow: 0.6 kg m^−1^ yr^−1^	At low flow: 23%	[228]	3 years
Arkansas, USA; 35 ha basin (simulation)	Experimental vegetated surface drainages 60 m length	“rice spill” weirs	TIP	10	0.02 kg m^−1^	86%	***[235]	7 days x 2 trials
			Nitrate	2–15	0.4–0.6 kg m^−1^	97%		
		Open flow Riser pipes	TIP	10	0.02 kg m^−1^	88%		
			Nitrate	2–15	0.4–0.6 kg m^−1^	79%		
		Low-grade weirs	Nitrate	3–4	>1.2 kg m^−1^ yr-^1^	79%	[236]	8 hours
Southwest Sweden; Each plot 0.2 ha	Subsurface, experimental plots of potatoes	enclosed riser pipes, ~ 90–130 cm above free flow	TP	-	>0.028	58–85%**	[209]	22 months
			Nitrate	>11–19	>30–38	78–94%**		
		Controlled drainage 20-70 cm below surface	TP	> 0.02	>0.026–0.138	56–95%**	[237]	4 years
			Nitrate	> 9–10	>26–37	69%–94%**		
Northeast Italy; 0.001 ha plots	Subsurface, experimental plots of beets, maize, or wetland plants	Controlled drainage 0–60 cm from surface	Nitrate	>8–77 (upper range)	3–11.8 g m^−2^	46%–63%**	[225]	31 months
		wetland				96%**		
Ontario, Canada; 1.9 ha plots	Subsurface, maize	Riser <60cm from surface	Nitrate	>19.2	58	46%**	[238]	1 year
Ontario, Canada; 4 ha plots	Subsurface, soybeans	INNOTAG controlled drainage units <65 cm from surface	Nitrate	12–15	>16.9	14%–25%	[239]	2 years
Ontario, Canada 0.1 ha plots	Subsurface corn/soybean	Riser 30cm above free flow	Nitrate	>4–8	>1.7–19	31%–44%	[240]	4 years
	Subsurface + subirrigation, corn/soybean					62%–66%		
Lithuania; 4.9 and 5.4 ha plots	Subsurface; barley, winter wheat, summer wheat, rape	Riser 68cm above free flow	Nitrate	5–25 (total range)	>14	22%	[241]	7 years
Ohio, USA; 12 plots, each 0.066 ha	Subsurface; corn/soybean	Riser 30 cm below surface;	Nitrate	>9.5–16	>14–24	45%	[226]	4 years
	Subsurface +subirrigation					30%		

* If inflow loads or concentrations were not reported, values are listed as > outflow. Loads are estimated from discharge and concentration in water, rather than terrestrial fertilizer application; ** Compared to conventional drainage, other % is based on inflow *vs.* outflow; *** 2 mg/L NO_3_^−^ - N ditches acted at N source, DIP values reported in Table 2 of original document are actually TIP due to typographical error.

In ditches that have at least a modest elevation gradient, low-grade weirs placed in series [198] can essentially create a mosaic of instream wetlands in agricultural ditches [242]. While individually these wetlands may be small in relation to their watersheds, their cumulative effect can be substantial, both with respect to biodiversity and nutrient removal [243]. A summary of case studies of agricultural wetlands demonstrated substantial nitrate reduction in wetlands that were less than 0.5 hectares and with wetland: watershed ratios as low as 1:180 [79]. Load reductions of total nitrogen for various configurations of small instream wetlands are regularly reported as 30% or higher, even for wetlands that constitute only 1% of the total drainage area [244,245,246,247,248,249]. High loading rates that coincide with low temperatures, however, greatly decrease the effectiveness of these wetlands, resulting in maximum removal rates of as little as 15% [250]. 

Wetlands are generally good sediment traps, but when hydraulic retention times are only a few hours, finer sediments and associated nutrients already suspended in the water column are likely to remain so, whereas fine sediments deposited during low flows can be re-suspended [251]. The reducing conditions common in wetlands can release dissolved phosphorus and ammoniacal nitrogen from the substratum, adding to any particulate nutrients suspended in the water column, net increases in total phosphorus, or even nitrogen may occur [246,247,248]. Although instream agricultural wetlands that are less than 1% of the watershed area can effectively remove approximately 20%–40% phosphorus [251], greater relative wetland area, around 5% of the total drainage area, act more consistently as phosphorus sinks [248,249]. 

Although the studies cited above give the impression that smaller wetlands would be preferable for nitrogen removal, whereas larger wetlands would be better for phosphorus removal, due to the plethora of other factors affecting load reduction rates, the issue is unclear. Tomer *et al.* [252] in a non-technical discussion of using wetlands for nonpoint source pollution suggest that larger wetlands are, in fact, better for nitrogen removal. This apparent discrepancy is related to issues of scale, hydrology, and source distribution. The wetlands discussed above are small and have little riparian buffering. Larger wetlands located at stream confluences tend to have more riparian vegetation and greater hyporheic exchange, resulting in more overall denitrification. Also, in many agricultural wetlands nitrogen is primarily found as labile nitrate, while phosphorus is closely associated with sediment transport. Thus multiple small wetlands strategically located downstream of highly erodible areas would be expected to retain phosphorus, whereas the low hydraulic retention times may be insufficient for substantial nitrate reduction. This trend would likely be more pronounced in systems where very high rates of subsurface flows at the field-scale limit interactions with plant roots and denitrifying bacteria. 

In comparison, under exclusively surface-drainage, short-term studies in experimental ditch systems have demonstrated the potential for high nitrogen sequestration using multiple instream structures to create wetland conditions, as opposed to a single outlet control using risers [235,236]. Such increases, however, varied depending upon nutrient concentrations, duration of high-load events, and preceding hydrologic conditions. Regardless of whether water was held at multiple structures or a single structure, load reductions were generally above 80% for nitrate and total phosphorus. Investigations are currently underway to assess the efficacy of similar structures for remediation of a wide range of nonpoint source pollutants on active farms [253]. 

**Figure 6:**
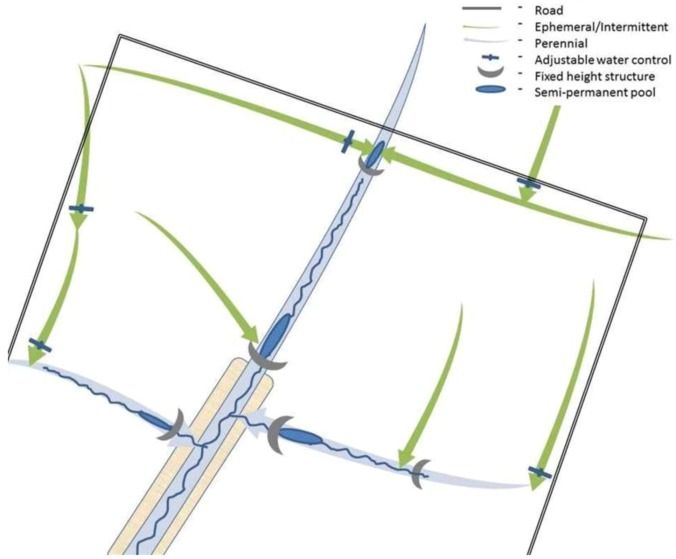
Schematic showing a hypothetical hierarchical arrangement for in-stream practices at the field scale (approximately 0.25 hectares). Ditch and structure width is exaggerated for illustrative purposes. Small, adjustable water control devices, such as the flashboard risers or slotted concrete weirs are situated in smaller ditches along the perimeter road for easy access. Non-adjustable low-grade impediments are placed at regular intervals along the reach and upstream of confluences. These devices can be log vanes, armored berms, or simply artificial riffles. Ideally, they are placed downstream of shallow pools and empty into a two-stage or naturally meandering system.

Often, primary reaches in headwater ditches already possess the defining characteristics of wetlands: shallow inundation during part of the growing season, hydric soils, and hydrophytic vegetation. The primary difference between these “ditch wetlands” and more familiar classifications is the high level of disturbance, resulting from both extreme hydrology and direct management practices. Methods that include multiple site-specific practices in a watershed context serve to dampen variability in hydrology and stabilize geomorphology in highly disturbed systems, while increasing habitat quality and allowing a focus on specific impairments in more stable systems (Figure 6). Although decreasing these potential disturbances and sources of environmental stress are prerequisite for establishing ecosystem function, a more comprehensive approach based in ecological principles is necessary for this potential to be realized [11,30,84,254]. 

An ecosystem-based approach; however, must take into consideration the interaction of physical, chemical, and biological factors. There has been little research directed toward how such structures affect habitat in agricultural headwaters. As previously mentioned, the ponding of water resulting from erosion control structures in streamside gullies has been demonstrated to increase biodiversity of those systems [190,191,192]. The effects of instream water control structures, however, function differently from the drop-pipes used in those studies, and the gullies were essentially depauperate of aquatic and wetland animals prior to implementation. Research has demonstrated localized improvements in macroinvertebrate habits associated with the rip-rap commonly used for grad control structures in agricultural headwaters [255]. Additionally, structures such as low-grade weirs may increase the number and depth of pools, which has been advocated for improving fish habitat in agricultural drainage systems [143]; however such structures may require modification to allow fish migration [256]. As stream order and/or biotic integrity increases, the utility of such structures for sediment and nutrient retention is likely at odds with the potential detriments to aquatic habitat [257]. 

This contrast highlights the difficulties of reconciling the multitude of desired outcomes of environmental restoration. The following section examines some of the interactions related to management, land-use and biodiversity in agricultural headwaters in the context of ecosystem functioning and stability, specifically focusing on macrophytes. Because the impacts of agriculture on aquatic systems are related to the area of the watershed in agricultural production, small streams inset within or immediately adjacent to agricultural fields are expected to have relatively lower biodiversity and “natural” functioning than streams draining larger watersheds. Although many of the concepts discussed are applicable to a broad array of ecosystems, these highly degraded and potentially more manageable systems are the primary focus.

## 5. Ecosystems and Communities

The term biodiversity is inclusive of several inter-related concepts such as species richness, genetic heterogeneity, and functional traits related to the behavior of organisms and the resulting effects on their surroundings [258]. Rather than attempt to elucidate the differences between these concepts, a more utilitarian approach in the context of managing drainage systems is to address their commonalities. This approach relies on the idea that, whether the hierarchical level of diversity is genetic variability within a population or degrees of trophic interactions within an ecosystem, diversity is proportional to biotic resilience and niche utilization/creation [259,260]. The validity of this premise is debatable, but it is based soundly on basic probability and concepts that are widely accepted in the biological sciences [261]. One of the basic tenets of natural selection is that organisms within a species vary in their general behavior and in their specific responses to environmental factors. The same property applies when comparing different species, functional groups, trophic levels, *etc.* [262]. 

Increased biodiversity—in the general sense—increases both the potential range of organismal responses to environmental change and the functional redundancy among groups of organisms. So, whereas individuals, or even species, may come and go in response to change; unless the change is cataclysmic, if a sufficient population of other individuals or species are present there is the possibility that they can fill the same broadly defined ecological role. For example, in a stream reach with only one type of snail, a pathogen could cause complete extirpation, leading to increased epiphyte growth and subsequent loss of the macrophytes that provide the habitat for a broad array of organisms. In a system with several types of snails, there is an increased likelihood that another type would increase its population in response to increased resources, thus buffering system-level change. More generally, the probability that a given system disturbance would have major influences on system function is decreased. The effects of biodiversity on ecosystem resilience and on ecosystem services and functions related to improved water quality represent an emerging topic of great practical importance with regard to ecological restoration and rehabilitation [14].

### 5.1 Spatiotemporal effects on macrophytes

Following this same logic, it is argued that increasing habitat heterogeneity correspondingly increases biodiversity via increasing the availability of niches [263]. This heterogeneity may not be obvious via casual observation. Relatively high biodiversity observed in a ditch in the United Kingdom was partially attributed to a concurrent gradient in pH [264]. Heterogeneity, however, can be a double edged-sword, as maintenance of a viable population can be less likely in smaller patches of habitat than in larger patches [265] meaning that relative scales of heterogeneity are an important consideration. For example, the occurrence of functional traits, such as denitrification, among microbes can be fairly diverse in ditches that appear homogeneous [266]. Furthermore, in environments with multiple sources of physiological stress, those stresses can be more important than arbitrary measures of heterogeneity for limiting diversity and biotic assemblages [267].

That being said, in reference to agricultural ditches, increasing some types of heterogeneity is likely to increase plant biodiversity, especially in systems where hydrology and poor substratum are a major source of stress. Two-stage ditches and water control structures that decrease extreme temporal variability also increase spatial heterogeneity of both substratum and hydrology. Stabilization structures, whether geologic or biotic in nature, not only decrease rates of change in channel morphology, they also serve to add stable surfaces for attachment of organisms. Artificial riffles and low-grade weirs armored with rip-rap may have the added benefit of creating deposition zones of soft sediment suitable for rooted macrophytes and burrowing invertebrates. 

Even at small scales, increasing the available volume of soil or sediment that roots can penetrate increases plant diversity [268]. Riparian plant species richness is related to increased width of the sloping banks [269], which correspondingly increases available habitat and likely increases the probability for water-dispersed seeds to settle on the banks [180]. The ecology of aquatic and wetland plants within agricultural drainage ditches is an emerging field relying heavily upon principles of landscape ecology as well as wetland and stream ecology. Some of these principles and the studies elucidating them are explained below. 

**Figure 7:**
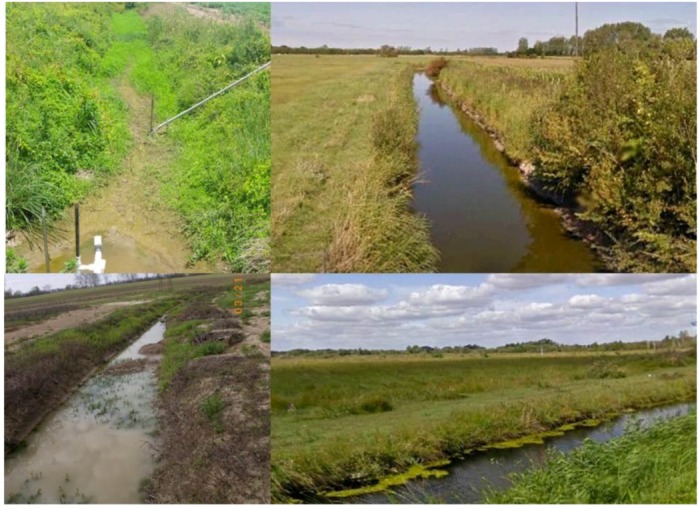
Ditches in different parts of the world have many characteristics in common, but differences in hydrology impact vegetation. *At left*, seasonally inundated ditches near Yazoo City, Mississippi, USA. If the channel bed is fairly stable ditches like these allow growth of emergent vegetation, which becomes lush as water recedes during dry summer months. *At top right*, ditch draining pasture land near Sainte-Mere-Eglise, Normandy, France. Note that although the water is much less turbid than the ditch near Yazoo City, emergent vegetation is limited to the bank due to persistent inundation. *At bottom right*, ditch in Wicken Fen, Cambridgeshire, U.K., likewise lacks emergent vegetation. Although this area has been managed as a nature reserve for over thirty years, mats of floating vegetation are clearly visible on the water’s surface.

In an experimental study in a wetland with fairly constant water levels, small substratum elevation differences within single plot resulted in increased measures of diversity in wetland plant assemblages [270]. Similar patterns have been observed in other studies [271]. In addition to potential differences in water availability, differences in soil oxidation-reduction chemistry between inundated and drained soils invoke species-specific stress responses to oxygen depletion and change the availability of plant nutrients and toxins [272]. Because of stresses related to oxidation-reduction conditions in the soil, plant response to water depth and wetland hydroperiod are a major determinant of dominant vegetation (Figure 7, [273]), especially during establishment [274]. 

In lotic systems, water velocity can be an important determinant of plant assemblages [275,276]. The creation of multiple zones of different hydrology, such high velocity riffles, low velocity pools, and frequently inundated benches, increases physical habitat diversity, which allows for the persistence of organisms with a wide range of tolerance limits, and potentially increases biological diversity [277]. In reaches of highly variable hydrology, small, permanent pools of water may be especially important in preserving local aquatic diversity in conditions of seasonal drought and uncertain climate patterns. Relative to their size, for example, small ponds have been demonstrated to contribute substantially to overall biodiversity in areas of extensive agriculture [278,279]. Maintaining permanent pools of water in ditches, or at the very least areas of saturated soil, is especially important if larger wetland and aquatic systems are few or distant [84]. 

In areas of extensive agriculture, long-term decreases in macrophyte species richness have been observed [27,28,29,30,280]. These decreases are due not only to hydrologic alteration and non-point source pollution, but also due to landscape factors affecting plant dispersal [281]. In frequently disturbed or highly fragmented environments, species colonization plays a major role in determining community composition [140,282]. Localized extirpation of macrophytes in small headwater ditches may result from greater environmental stochasticity [278], extreme physicochemical conditions [17,278,283,284], and higher anthropogenic stress and disturbance [17]. 

A major potential source of disturbance is removal of vegetation from ditches and banks as a standard drainage maintenance practice [283,285,286,287]. Maintenance of ditch banks often entails regular mowing or herbicide application and infrequently uprooting of woody vegetation and re-sloping of ditch banks. Channel dredging, performed on an as-needed basis may remove large areas of channel vegetation along with accreted sediments. For the resulting bare earth to provide any degree of structure or habitat it must be re-vegetated. Unless ditches are under active conservation management that includes a seeding or planting regime, this reestablishment must take place via vegetative expansion of any viable plant tissues on site, from the seedbank, or from dispersal from adjacent habitat [27,288]. Both seedbanks and nearby habitat for source populations have been demonstrated to influence ditch macrophyte communities [27,178,282,289]. Riparian systems, with their extensive habitat connectivity and resulting biodiversity can serve as sources for repopulation of habitat disturbed by agricultural management [178,264]. The high degree of environmental variability and disturbance in agricultural ditches, however, generally precludes establishment of plant assemblages with comparable composition to their likely source assemblages [288,290]. 

In spite of the importance of dispersal for understanding ditch plant assemblages, in comparison to studies analyzing source-sink dynamics from landscape or metapopulation perspective, there has been relatively little research elucidating modes of ditch plant dispersal. Blomqvist *et al.* [27] found that in species whose occurrence on ditch banks was increasing, the most common mode of dispersal was related to agricultural activities, whereas water dispersal was relatively unimportant. Other studies examining dispersal methods have concurred that farm machinery can be an important mode of dispersal, though dispersal by wind and water are also substantial [281,282,291]. It is important to note that these studies are concerned primarily with terrestrial or facultative wetland plants occurring on the ditch bank, rather than in the channel. Dispersal via water can be an important mode for wetland plant colonization of ditches having perennial flows of low velocity, and appear to be influenced by wind patterns, potentially allowing colonization of upstream habitats [180]. In ephemeral or intermittent reaches it is unlikely this “sailboat” method of dispersal would occur, underscoring the need for maintaining small pools of water for local seed production and only removing vegetation from ditches in areas where it is expected to cause flooding.

### 5.2 Microhabitat Effects on Macrophytes

Although availability of propagules can be an important source of plant diversity, in an agricultural setting, microhabitat factors can result in plant assemblages that are very different from those in the available seedbank and likely source assemblages (Table 3, [27,290]). In areas that are subjected to regular inundation, plant species richness is often a function of abiotic factors, rather than interactions among organisms, such as competitive exclusion [292]. The most important of these factors are related to hydrology. For example, plant assemblages in meadows develop around niche separation based on soil moisture patterns [293]. As mentioned previously, relative water depth can be an important determinant of plant assemblages and plant species richness [270]. In areas of variable hydrology, however, the intensity and duration of flood events are likely more important for determining plant assemblages than depth at any one time [294]. In the context of ditch rehabilitation, management related to frequency and duration of high water events can influence plant composition [295]. 

At the other end of the hydrologic spectrum, low water retention can result in episodic droughts leading to extirpation of wetland plants used for restoration [296,297,298] and may be related to the dominance of annual facultative species in some ditch reaches [17]. Use of controlled drainage practices to raise water levels has positive benefits on ditch vegetation in perennial low-flow ditches [299]. By increasing local hydroperiods, such structures would also increase the likelihood of persistent wetland vegetation in ditches subject to episodic drought. Because the location of instream structures is a function of stream gradient, increases in hydroperiods would ideally be limited to a small adjacent area, allowing for organisms adapted to ephemeral hydrology to persist over less influenced reaches of the stream. Controlled drainage also has the potential to decrease peak discharge rates of receiving waters. Although the relationship between macrophyte abundance and discharge/velocity has not received the same amount of attention as other hydrologic parameters, the relationship is likely a dominant force in lowland streams [275]. In general both abundance and diversity of macrophytes is limited by high velocities [300]. Few studies have examined these effects in agricultural ditches, but Schaller *et al.* [301] observed decreases in plant biomass following high precipitation events.

These stressors not only influence the diversity and coverage of plants, but also influence the basic growth forms of plants that can survive. The most basic growth form categorization of macrophytes describes the position of photosynthetic parts of the adult plant in relation to the water surface. Submersed (or submerged) plants can survive using exclusively underwater photosynthesis, acquire a large portion of mineral nutrition directly from the water column and have a root system that is often primarily for anchorage. Floating-leaf plants rely upon contact with the atmosphere for photosynthesis and may be unanchored, anchored to the channel, or have an architecturally simple root system connected to the channel bed or shoreline. Emergent plants likewise maintain contact with the atmosphere, but are more intimately associated to the substratum by extensive root systems and often rhizomes. 

Generally, submersed macrophytes are not prevalent in agricultural headwater ditches. High hydrologic variability and mechanical shear stress resulting from high peak discharge and high turbidity due to nutrient enrichment or suspended sediment preclude establishment and growth [109,275,302]. These trends toward decreased occurrences of submersed plants in drainage ditches may be the result of eutrophic compression of the photic zone, as is often considered the case in lentic systems. In agricultural headwater ditches, however, hydrologic variables related to discharge and velocity are a predominate factor that can limit submersed and floating-leaf vegetation [275,303]. These stresses, along with dispersal characteristics, likely explain why floating leaf macrophytes in agricultural landscapes are generally more common in higher order streams [304], or ditches with a fairly stable hydrology [180,289], than in small streams and ditches with stochastic hydrology [17,278].

Headwaters of agricultural drainage ditches are often dominated by emergents, particularly grasses [305] such as *Leersia* spp. [17], which have a seedbank that can persist under inundation. Although it is an oversimplification of a broad taxonomic group, the growth habit of rhizotomous grasses and the fact that occurrence of grasses in drainage ditches is related to high nutrients [306] mean that they have the greatest general utility for initial restoration or rehabilitation efforts in lowland agricultural environments [307]. In general, emergent macrophytes that persist in the harshest ditches are tall enough to maintain leaf contact with the atmosphere during flooding or can withstand periods of submergence in turbid water. The latter strategy requires some type of storage tissues, such as the rhizomes used by grasses and the grass-like *Typha* species. Energy storage via rhizomes is also important for re-sprout following burial due to sedimentation or bank sloughing. 

**Table 3:** Localized habitat parameters affecting macrophytes in wetlands and streams in the agricultural landscape. Note that these factors also indirectly affect macrophytes via changes in community structure of consumers and decomposers.

Causes	Effects
Lack of canopy	High temp, increased primary production
Increased peak discharge	Mechanical stress, temperature variability
Increased hydrologic variability	Drought/anoxia stress
Increased slope of ditch bank	Burying, sharp gradient limits area for establishment, shading
Suspended sediment	Turbidity, scouring
Elevated N and P	Turbidity, increased primary production
Substratum	Less variability, compaction, unconsolidated fine particulates
Herbicides/Pesticides	Plant toxicity

Other plants with similar growth habits, such as species of *Polygonum* that can spread clonally and have a fairly fibrous root system are also common in such ditches [17]. During storm events, high velocity water that is laden with sediment scours both the surface of the plant and the substratum. Conversely, during periods of drought, plants growing in fine mineral sediments must cope not only with decreased water availability, but also mechanical stresses on roots due to contractions and subsequent cracking during drying. In both extremes clonal plants with interconnected rhizomes can be important for structural stability, essentially weaving a living mat of plant material. In relation to ecological function, these types of plants can be broadly categorized as “matrix” species, as they tend to be influential in shaping the general structure of a wetland [308]. Other taxa that include such plants are *Scirpus, Typha*, and *Phalaris*, which are all commonly used plants in treatment wetlands. In contrast, plants that form isolated tussocks may be limited to ditch margins or pools created by water control structures. The importance of such plants should not be discounted; however, as the microtopographic heterogeneity provided by the tussocks can be an important source of plant diversity following senescence [309]. 

### 5.3 Ecosystem Functions of Macrophytes

Issues related to vegetation cover, type, and persistence in ditch systems are important because plants form the structural base upon which the ecosystem is built [310]. Vascular plants directly or indirectly regulate a number of processes in a variety of aquatic ecosystems. Benthic macroinvertebrate abundance, for example, is related to increased macrophyte cover [311]. In open canopy systems lacking woody debris and course substratum, such as cobble, disturbance to macrophytes results in changes in macroinvertebrate assemblages that can cascade through multiple trophic levels [312]. Submerged portions of plants serve as growth surfaces for bacterial biofilms and epiphytic algae [313,314,315], which are the basis of most aquatic food webs. Additionally they serve as habitat for a number of aquatic organisms [316]. Within drainage ditch systems, higher macrophyte diversity has been associated with greater abundance and species richness of animals [264], but no clear cause-and-effect relationship has been established. Although generalizing these complex interactions is difficult, they depend to some degree on plant growth form. 

For example submerged and floating macrophytes displaying a high degree of structural complexity in the water column can increase the abundance and diversity of macroinvertebrates [108,316,317]. Conversely, in wetlands with persistent inundation, dominance of emergent macrophytes can also result in lower overall macrophyte diversity in comparison to systems dominated by submergents [318]. Plant growth-form also affects non-point source pollution and related functions [319,320,321,322]. Plant surfaces in the water column cause drag [323], which decreases velocity and increases retention time [324], inducing sedimentation [325] and decreased resuspension [326]. Marginal emergent vegetation, however, can eventually increase stream velocity [175], due to stream narrowing, as previously discussed. Surveys examining both vegetation and nonpoint source pollutants in agricultural drainage systems have noted inverse correlations between vegetative cover and concentrations of suspended solids [17,327]. The efficacy of plants for decreasing suspended sediment depends not only upon the amount of vegetation, but also the growth-form. In comparison to submergents, emergent vegetation shows greater reductions in sediment resuspension [300,328]. Likewise, because plant roots stabilize bank and bed stability via physical structural reinforcement and pedogenesis [91,212,329,330] emergent plants with their more extensive root systems are more often used for increasing structural stability. 

Although the effects on plant-induced decreases of nutrients are well documented [131], comparative studies have also reported lower loads of non-point source pollutants in ditches lacking vegetation [331], or in which the aboveground biomass of vegetation was recently removed [332]. The complexities of phosphorus removal and plants are especially vexing, due to the sometimes opposing effects of plant-induced changes in pH, organic carbon, and oxygen availability in both the water column and the substratum. The way plants impact nitrogen and phosphorus are very different. Take organic assimilation for example. Compared to nitrogen, which can constitute up to 3% of dry mass in wetland plants, phosphorus is generally below 0.5% [333]. Because most of the phosphorus in flowing agricultural waters is particulate bound, the majority of total phosphorus removal from the water column by plants is probably due to sedimentation. In waters with high levels of particulate nitrogen [334], this path may also result in nitrogen being removed. There are also indirect pathways in which plants can impact nutrients in the water, such as altering pH or oxidation-reduction potential in the water column as daily photosynthesis/respiration cycles alter concentrations of oxygen and different species of inorganic carbon [335]. 

Wetland and aquatic plants also alter soil and sediment chemistry [323,336,337,338]. Two well-established examples are the release of oxygen [272] and organic carbon [339,340], which essentially create biogeochemical hotspots. These hotspots may immobilize phosphorus [335,341,342]; however, higher concentrations of organic phosphorus have been associated with plants [343]. Furthermore, as there is no major gaseous phase in the phosphorus cycle, these immobile forms remain in the system and may be released into the water column when plants senesce [197,343]. In systems with non-calcareous mineral substrata much of the immobile phosphorus is associated with aluminum and iron [344]. Theoretically, inundation in these systems can neutralize pH and reduce ferric phosphorus complexes, leading to dissociation and increased solubility of phosphorus [130]. If pH is already close to neutral, plant-induced soil acidification would potentially attenuate this release, but at a range of pH3–4, phosphorus can dissociate from aluminum, increasing concentrations in the water column [130]. With regard to drainage ditches, the effects of management further complicate matters. Simply removing vegetation and sediment from ditch beds can result in complex responses in phosphorus transport that vary over time [345,346]. 

Unlike phosphorus, reduced nitrogen species readily volatilize; thus denitrification of nitrate represents a potential permanent removal mechanism. Because of high nitrate concentrations in agricultural headwaters, denitrification rates are often limited by the availability of organic carbon in the sediment [172,347]. Thus the organic carbon that plants release into the oxidation-reduction gradients around flooded roots would be expected to increase denitrification and may partially explain why plants are associated with higher rates of denitrification and lower concentrations of nitrate in agricultural streams [327,348]. In the highly productive vegetated edge-of-field ditches in the LMAV, for example, denitrification potential during the growing season can be comparable to constructed wetlands and natural depressional wetlands [349].

Not surprisingly, the impact of plants on oxidation-reduction conditions of the substratum also differs among species, potentially affecting effluent nutrient concentrations [350]. More generally, studies have demonstrated that floating-leaf macrophytes and emergent macrophytes promote greater denitrification rates than submersed plants [318,351]. The increased denitrification by floating-leaf macrophytes is likely caused by decreased light penetration limiting oxygen production in the water column and benthos, creating an environment more conducive to chemical reduction. Emergent plants also shade the water column, but their primary impact on denitrification is more likely the result of the chemical conditions they create in the rhizosphere and the benthos in general. 

Although denitrification is generally considered the primary pathway for nitrogen removal in agricultural effluent, it is important to note that denitrification is not the only pathway. When the actual nitrogen removal rates for different growth forms of aquatic and wetland plants are compared, a pattern emerges that is very different from that observed for denitrification. Generally, emergent plants are least effective for removal of dissolved nutrients such as ammonia, nitrate, and soluble orthophosphate. In comparisons to emergent macrophytes, Srivastava *et al.* [320] estimate that floating-leaf rooted macrophytes are five times more effective, free-floating macrophytes are six times as effective and submerged macrophytes are nine times as effective. Due to the aforementioned high levels of stress and disturbance in these headwaters, even under intensive management maintaining extensive submerged or free-floating macrophytes would be difficult. Some species, such as the water hyacinth, *Eichhornia crassipes*, form dense colonies of interconnected surface mats that allow some degree of anchoring under flow. Mats of this species have been reported as removing 777 mg N m^−2^ d^−1^ and 200 mg N m^−2^ d^−1^ (or 280 g N m^−2^ yr^−1^ and 73 g N m^−2^ yr^−1^, [352]). 

### 5.4 Potential Impact of Aquatic Macroinvertebrates on Nutrient Export

Although drainage management practices are likely to decrease export of nitrogen, suspended sediment, and associated particulate phosphorus, over the long term, phosphorus export may continue to be a problem due to leaching of dissolved phosphorus [168]. Because the volatilization of phosphorus is insignificant under conditions found in drainage ditches, removal via biogeochemical processes similar to nitrogen reduction are not possible. Plant uptake of phosphorus in ditches dominated by herbaceous plants is also not a long-term solution, as plant-related phosphorus removal decreases with system maturity and phosphorus assimilated by plants may be released upon senescence [197,343]. Accretion of particulate-bound phosphorus in sediments is often presented as a viable method for decreasing phosphorus loading to receiving waters. This approach, although useful, can lead to internal loading of dissolved phosphorus. 

Furthermore, in areas where a large portion of particulate bound phosphorus is associated with iron or aluminum, the effects of inundation on pH and oxidation-reduction conditions can cause dissociation, leading to elevated concentrations of soluble orthophosphate in the water column. This process can be enhanced under conditions of drying and rewetting, which also promotes the mineralization of organic carbon, increasing phosphorus solubility [131]. Thus in the long-term, while phosphorus loading to receiving waters may be decreased via sedimentation, levels will likely continue to be elevated due to the continued internal loading and influx of dissolved phosphorus from near-saturated terrestrial soils. Some degree of internal loading may be curbed by removal of sediment as part of standard dredging practices. However, extensive dredging of phosphorus-saturated sediment can lead to increased export of suspended sediment, which is counter to the desired effect. A largely unexplored approach is to manage ditches for biodiversity as a means to increase biotic export of nutrients to terrestrial environments. 

Nutrient exchanges between aquatic systems and their watersheds have been described as highly asymmetric, with the terrestrial environment providing nutrients and the aquatic environment receiving [353]. The classic model of riverine ecosystems assumes little instream primary production in headwaters [171]. As previously discussed, however, this model rarely applies to areas of extensive agriculture where ample light and high input of mineral nutrients can result in extensive algal blooms. Research demonstrates that even in shaded, mesotrophic streams some functional groups of insects feed almost entirely on instream algae [353]. The fact that aquatic insect larvae may preferentially utilize autochthonous materials is important because the majority leave the aquatic environment upon reaching maturity, taking with them any assimilated carbon, nitrogen, and phosphorus. A growing body of research suggests that insect emergence represents a major energy flux from aquatic to terrestrial systems [354,355,356,357,358]. These studies, as well as those cited below, have been primarily concerned with the ecological ramifications to terrestrial systems, rather than the potential effects on instream nutrient cycling. 

Stoichiometric analyses of insects show they are often up to 1% phosphorus [359] and 10% nitrogen [360]. In comparison, an aquatic system can be classified as hypereutrophic with as little as 1 × 10^−5^% phosphorus and 1.2 × 10^−4^% nitrogen in the water column [164]. After taking into consideration that organisms accumulate these nutrients at concentrations several orders of magnitude greater than environmental concentrations, it becomes apparent that biotic fluxes of limiting nutrients moving from aquatic to terrestrial systems may represent an unexplored method for nutrient mitigation in highly disturbed, biotically depauperate systems. The most striking example of the potential for biotic export is a study by Jackson and Fisher [354], wherein they calculated a net biomass export of over 20 g m^−2^ yr^−1^ from a temperate desert stream. Using the stoichiometric values above, the calculated export of nitrogen would have been approximately 2 g m^−2^ yr^−1^ and phosphorus would have been 0.2 g m^−2^ yr^−1^. For a reach 1 km long and 1 m wide the annual removal rate would be 2 × 10^2^ g of phosphorus and 2 × 10^3^ g of nitrogen. Other studies have found emergence rates approaching an order of magnitude lower (e.g., 3.7 g m^−2^ yr^−1^ in the 14 lotic systems reviewed by Jackson and Fisher [354]. Thus associated export would likely be correspondingly lower. These removal rates are insignificant compared to the wetland plant uptake values estimated by Kadlec and Wallace [131], which are approximately 129 g m^−2^ yr^−1^ for nitrogen and 6 g m^−2^ yr^−1^ for phosphorus. However, because insect emergence represents a nutrient export from the aquatic system, rather than internal accumulation, it removes nutrients from the aquatic system, rather than just from the water column. 

For biotic nutrient export to be examined, first it is important to catalogue assemblages of insects and other macroinvertebrates that occur in ditches and to explore what might be limiting their diversity and productivity. Although macroinvertebrates represent the most visible link between primary producers and economically important fisheries in receiving waters, like most aquatic fauna, their role in highly disturbed agricultural headwaters has received little attention. A few examples, however demonstrate that hydrology may be a principle driver of these assemblages. In a study of drainage ditches in California, macroinvertebrate density was generally low, and dominated by pollutant tolerant gastropods, oligochaetes, and invasive *Corbicula* clams [361]. The authors point out that these low numbers are likely a result of poor substrate and ephemeral hydrology, as perennial streams are usually more biodiverse than non-perennial streams [362,363,364]. A survey of benthic invertebrates in Arkansas showed similar trends with ephemeral headwater ditches displaying low aquatic insect diversity and density [365]. It is unclear whether these trends are the result of immediate anthropogenic influences related to agricultural management, such as pesticide use, or simply a response to hydrology and substrate changes corresponding with stream reach. Although it is conceivable that the trend is related to nutrient enrichment, in general, streams high in nutrients will display increases in invertebrate production [366]. A survey of ditches in the Netherlands found macroinvertebrate diversity in ditches comparable to that in small lakes [367]. A similar survey in Florida found higher macroinvertebrate diversity in ditches that were hydrologically connected with streams that had undergone hydrologic alteration than in natural streams [181]. In both these systems hydrology was comparably stable and pesticide exposure was likely lower than in the ditches in Arkansas or California. 

In addition to nutrient fluxes due directly to insect emergence, fluxes can occur via other biotic vectors. Amphibians, for example, may represent a net efflux of nutrients in aquatic systems, as within a cohort, the overall nutrient loads from egg masses are generally lower than loads exiting the system upon emigration [368,369]. High mortality due to insufficient hydroperiod, however, can reverse this trend [370]. In the context of agricultural headwaters, other potential stresses and disturbances could result in mortality rates comparable to those observed by Register and others [371,372]. Increasing trophic levels and food web connections within aquatic systems can also have positive benefits on nutrient mitigation. Vanni [373] cites several studies that demonstrate that long-lived aquatic organisms, such as mussels and fish, may represent a significant long-term, albeit temporary sink of nutrients. Simulations performed by Small *et al.* [374] also suggest that instream consumers can greatly influence nutrient cycling, potentially remediating loading of critical nutrients like nitrogen and phosphorus. Admittedly, diverse aquatic communities comparable to those in less impacted streams are not a likely outcome or realistic target for many primary agricultural drainage systems. If, however, diverse aquatic communities are to be preserved at all, rehabilitation must focus on headwaters and include multiple site-level practices incorporated into a watershed plan that considers ecological as well as physical processes [11,30,84].

## 6. Conclusions

Drainage for agricultural conversion is the primary cause for the degradation of wetlands and aquatic systems in many parts of the world. Impacts occur at multiple scales and include changes in the physical processes of ecosystems that broadly impact biota, generally decreasing biodiversity. Attempts at remediating these impacts, however, have suffered because they have ignored the overwhelming role of the initial interface between terrestrial and aquatic environments, which is most often the lowly drainage ditch. Efforts for improving the function of the land-water interface have generally been concentrated in wetlands adjacent to larger bodies of water and have taken a restoration approach that seeks to recreate some suite of historic conditions. Unless these projects include practices to mitigate the most distal headwaters, they are likely to fall prey to the same stressors and disturbances that they are supposed to remedy. 

New approaches focusing on these headwaters allow precision conservation practices utilizing a watershed approach. Because these approaches involve the cumulative effects of multiple practices, they are conducive to experimentation and/or adaptive management. Currently the focus of these practices is stabilization of hydrology and geomorphology and improved nutrient remediation, but they may have the added benefit of improving habitat conditions for aquatic and wetland biota. Such benefits, however, are reliant upon context of both landscape and management. In order to effectively manage environmental problems resulting from agriculture researchers and practitioners must not only develop practices that achieve arbitrary mitigation targets, but must understand how and why these practices work or how and why they fail. This understanding relies first upon the acceptance that artificial drainage ditches, rather than simply being physical conduits of water, are ecosystems similar to, but distinctive from, more familiar classifications of aquatic and wetland systems. As ecosystems that have been clearly demonstrated to impair receiving waters, artificial and highly impaired drainages in agricultural landscapes deserve no less attention with respect to their ecological functioning than the more biologically diverse systems they impair. 

### 6.1. Recommendations

Improving ecosystem functions and services in agricultural headwaters first requires site selection. Sites should be chosen first based upon their designation as critical source areas, or upon their impacts to critical downstream habitat. Rehabilitation efforts should be in proximity to large aquatic or wetland complexes, not only to decrease pollutant delivery to these systems, but also because they serve as source populations for aquatic and wetland organisms. In cases where agricultural streams and ditches drain uplands, the upland reaches should include riparian buffers, ideally forested buffers. If such buffers are not already in place, planting is a viable option, but in agricultural lowlands using subsurface drainage or with deeply incised streams, grass buffers or approaches that intercept concentrated flow paths are likely to be equally efficacious.

In the most severely degraded areas, rehabilitation should focus on system stability (substratum and hydrology) with biotic goals focused foremost on development of vegetative cover. Arrays of water control structures placed along reaches and starting at the most practical upstream point may improve vegetative establishment, attenuate hydrologic extremes, and create instream wetlands. Because they are effective for enhancing sedimentation, such structures are also useful when targeting projects specifically for decreased sediment and phosphorus transport. Such structures also increase hydroperiods in intermittent pools, potentially allowing colonization by non-emergent vegetation that decreases nitrogen via assimilation. However, implementation of control structures must consider a multitude of direct and indirect impacts on aquatic organisms. 

In reaches exhibiting perennial flow, stream-based approaches should be utilized whenever practicable to avoid unintended system-level changes. Both approaches should employ a strategy of creating multiple types of habitat by creating areas of different water depth or substratum composition to increase biodiversity and the probability of long-term habitat stability. In perennially inundated reaches with fairly homogenous depths of 0.5 m or greater, strategically timed periodic drawdowns may be an effective management technique for increasing diversity, especially in low velocity waters. If specifically targeting excess nitrogen, however, fewer species of the floating and floating-leaf macrophytes common in such waters may be more effective than a diverse assemblage of emergent plants and are less likely to impede flow.

Unfortunately, there is no catch-all approach for ecological restoration. Even somewhat simplified approaches attempting rehabilitation of ditches for a limited number of ecological functions require site-specific knowledge and modification. Thus these recommendations should really be viewed as considerations. Ultimately, there is no substitute for local expertise and these recommendations could have negative impacts in some situations. Some low gradient agricultural streams have a diverse array of mussels and crawfish that are dependent upon high velocity, low vegetation conditions, and would be exterminated by a high concentration of weirs. Subsurface controlled drainage, by decreasing discharge into surface waters, could elevate nitrate concentrations in groundwater to toxic levels, contaminating shallow wells. Furthermore, aquatic macrophytes are widely considered nuisances to be eliminated, rather than desirable organisms that provide services. The purpose of this review is not to serve as a how-to guide, but as a reference requiring a critical evaluation of how different practices apply to different situations. 

### 6.2. Future Directions

The path forward is not a straight line. Like an explorer paddling up a meandering river from its mouth to its source, each decision of which path to take will only lead to more choices as to which tributary leads to the desired destination. If this analogy can be applied to functional rehabilitation of aquatic systems, then the path forward relies upon a realistic expectation of the destination. Because headwaters drain small watersheds, and each watershed is unique, a quest for a definitive management protocol for these waters is more a matter of faith than science. Generally, though, with sufficient local knowledge, small watersheds may be more efficiently and effectively managed than regional watersheds. Additionally, the differences between smaller watersheds are easier to quantify, thus comparative approaches in multiple small watersheds would yield insights into relative effectiveness of management practices. If several of these studies were analyzed using spatially hierarchical models the cumulative effects of integrative management could be estimated. 

Robust meta-analyses, however, require standardized methods that are not widely applied in small watersheds, if at all. With regard to sediment and nutrient analyses, laboratory techniques have acquired at least some level of standardization, but sampling protocols vary, especially with regard to appropriate sampling intervals and areas. Given the inherent stochasticity of smaller streams in comparison to the more periodic variability in rivers and lakes, increased attention to sampling protocols is a necessary avenue of research. The issue of sampling protocols also arises when attempting to record usage of primary and secondary drainage systems by animals.

Studies examining such usage, generally measure occupancy as number of individuals, number of species, or living biomass. When ditches are located near aquatic systems in which anthropogenic impacts are low, occupancy values can be comparable to reference systems. The real question is whether or not the ditches are providing a habitat that contributes to a sustainable local population. In prime habitat, competition can lead to emigration of juveniles into less desirable locales, such as drainage ditches, increasing occupancy of these marginal areas. If the environment is not conducive to reproduction and offspring maturation, however, its habitat value is limited. Aside from a few studies documenting different growth stages of pollutant tolerant insects, data suggesting that ditches can support viable populations of aquatic animals are also limited.

In agricultural ditches draining row crops, conventional wisdom says that the majority of nitrogen removal takes place via denitrification of nitrate. If we ignore the possibility that other pathways such as biotic assimilation or anaerobic ammonium oxidation (anammox) are undervalued, there is still a major problem to be surmounted: benthic organic carbon limitation. Headwaters located in deciduous forests receive an annual resupply of organic carbon from litter fall, which is then trapped due to uneven surfaces and velocities. Even if existing ditches were planted with riparian trees, sufficient benthic carbon would likely decades to build up and would require some degree of channel heterogeneity to keep inputs from washing downstream during high precipitation events. More research should be directed at enhancing passive carbon input and creation of low maintenance bioreactors. Designs that can trap crop residues in the channel bed, but not cause flooding during high precipitation events, would be especially useful.

Because management practices will necessarily differ at different locations, it is important that case studies are available for areas of different geology, climate and agricultural practices. Referring to the Scopus search described in the introduction to agricultural impacts, the research is biased toward North American and Western Europe. In the broadly defined search using the word agriculture and either “environmental impact” or “ecological impact,” only 15% of the studies in this century were affiliated with researchers in Asia, 7% in Australia (including New Zealand), 4% in South America and only 2% Africa. Given the current area of land under agricultural production in China and India, and the increasing rate of land conversion to agriculture in equatorial regions, efforts toward applied research in reducing agricultural impacts in these regions should be paramount.

As research on this topic continues to expand and the relative environmental impacts of different water management strategies in different settings become more transparent, the perspective must shift from natural sciences toward social sciences. Only 4% of the studies found by Scopus in the search described above were categorized as social science studies. Although this discrepancy is to be expected to some degree, implementation of conservation practices may be a hard sell unless economic incentives or penalties are developed. Much of the land under intensive agriculture production is owned or managed by non-governmental entities that require some accounting of benefits before costs are accrued. Often, though, government sponsored programs will provide at least partial funding to encourage conservation practices. If such programs are to remain viable in the long-term, however, they must be demonstrated to have value to a variety of stakeholders and the general public.

The value of conservation programs can be demonstrated from a monetary perspective by estimating the potential cumulative derivatives of the ecological services they provide. Although this approach is gaining traction among some ecologists and economists, it subjects conservation efforts to an artificial economy wherein values are not based on supply and demand of tangible products. Also, the field suffers from a lack of standardization [375], further complicating interdisciplinary efforts. From the standpoint of justifying conservation practices, research on the valuation of ecosystem services is a necessary step. As a market-based incentive, however, it faces a number of ecological, political, and societal hurdles, as seen in the subjectivity of determining wetland mitigation credits in the United States, and the difficulties of wide-scale implementation of the comparatively simple system of trading carbon credits. In the 2012 grant cycle the United States Department of Agriculture, National Resource Conservation Service allocated over seven million dollars toward research for developing water quality trading credits. Actual nationwide implementation of such a system would require a substantially greater investment, which can only occur with support from stakeholders and the general public.

Ultimately, something is only worth what people are willing to pay for it. Any given individual in the general public is less concerned with how much an economic model says something is worth, than how much it impacts them directly. The conservation movement as a whole has relied upon charismatic animal icons such as elephants, tigers, and polar bears to elicit emotional reactions. For the majority of the population, this approach has the effect of removing conservation from their daily lives into a faraway exotic realm in which they are not active participants. Conversely, for those who live in close proximity with such animals, it creates a view of conservationists as out of touch with the realities of the dangers that are inherent to interactions between humans and the wild. 

Bringing conservation to the forefront of the sociopolitical dialogue requires broad-scale systemic and educational approaches as well as programs targeted specifically toward small stakeholder groups who have a disproportionate level of influence on environmental issues. Most conservation outreach, concerned with knowledge and perception among large populations, utilizes educational and sociological approaches to have the greatest effect on the most number of people. In contrast, outreach focusing on stakeholder groups such as farmers or agricultural consultants would be most efficacious using a behavioral approach geared at measuring the actions of individuals or small groups. The benefit of this approach is that the effectiveness of the outreach program can be experimentally quantified in concrete terms such as implementation rates. Either approach must rely upon not only short-term responses, but be designed to impact long term trends, as rehabilitation of aquatic systems will require permanent behavioral changes that create a multigenerational legacy of conservation practices.

## References

[1] Allan J.D. (2004). Landscapes and riverscapes: The influence of land use on stream ecosystems. Annu. Rev. Ecol. Evol. Syst..

[2] Blann K.L., Anderson J.L., Sands G.R., Vondracek B. (2009). Effects of agricultural drainage on aquatic ecosystems: A review. Crit. Revi. Environ. Sci. Technol..

[3] Carpenter S.R., Stanley E.H., Vander Zanden M.J. (2011). State of the world’s freshwater ecosystems: Physical, chemical, and biological changes. Annu. Rev. Environ. Res..

[4] Rabalais N.N., Turner R.E., Wiseman W.J. (2001). Hypoxia in the Gulf of Mexico. J. Environ. Qual..

[5] Breitburg D.L., Hondorp D.W., Davias L.A., Diaz R.J. (2009). Hypoxia, nitrogen, and fisheries: Integrating effects across local and global landscapes. Ann. Rev. Mar. Sci..

[6] Mander Ü., Kuusemets V., Hayakawa Y. (2005). Purification processes, ecological functions, planning and design of riparian buffer zones in agricultural watersheds (Editorial). Ecol. Eng..

[7] Strock J.S., Kleinman P.J.A., King K.W., Delgado J.A. (2010). Drainage water management for water quality protection. J. Soil Water Conserv..

[8] Kröger R., Thornton K.W., Moore M.T., Farris J.L., Prevost J.D., Pierce S.C. (2012). Tiered collaborative strategies for reducing hypoxia and restoring the Gulf of Mexico. J. Soil Water Conserv..

[9] Kröger R., Moore M.T., Thornton K.W., Farris J.L., Prevost J.D., Pierce S.C. (2012). Tiered on-the-ground implementation projects for Gulf of Mexico water quality improvements. J. Soil Water Conserv..

[10] Day J.W. Jr., Arancibia A.Y., Mitsch W.J., Lara-Dominguez A.L., Day J.N., Ko J., Lane R., Lindsey J, Lomeli D.Z. (2003). Using ecotechnology to address water quality and wetland habitat loss problems in the Mississippi basin: A hierarchical approach. Biotechnol. Adv..

[11] Evans R., Bass K., Burchell M., Hinson D., Johnson R., Doxey M. (2007). Management alternatives to enhance water quality function of channelized streams and drainage canals. J. Soil Water Conserv..

[12] Mitsch W.J., Day J.W. (2006). Restoration of wetlands in the Mississippi–Ohio–Missouri (MOM) River Basin: Experience and needed research. Ecol. Eng..

[13] Ranalli A.J., Macalady D.L. (2010). The importance of the riparian zone and in-stream processes in nitrate attenuation in undisturbed and agricultural watersheds—A review of the scientific literature. J. Hydrol..

[14] Brinson M.M., Eckles D.S.U.S. (2011). Department of Agriculture conservation program and practice effects on wetland ecosystem services: A synthesis. Ecol. Appl..

[15] Moore M.T., Kröger R., Moore M.T., Kröger R. (2010). Agricultural Drainage Ditches: Mitigation Wetland for the 21^st^ Century.

[16] Davies B.D., Biggs J., Williams P., Thompson S. (2009). Making agricultural landscapes more sustainable for freshwater biodiversity: A case study from southern England. Aqua. Conserv. Mar. Freshwater Ecosyst..

[17] Bouldin J.L., Farris J.L., Moore M.T., Cooper C.M. (2004). Vegetative and structural characteristics of agricultural drainages in the Mississippi Delta landscapes. Environ. Pollut..

[18] Moore M.T., Cooper C.M., Farris J.L., Lehr J., Keeley J. (2005). Drainage ditches. Water encyclopedia: Surface and Agricultural Water.

[19] Strahler A.N. (1957). Quantitative analysis of watershed geomorphology. Trans. Am. Geophys. Union.

[20] Beauchamp K.H., Pavelis G.A. (1987). A history of drainage and drainage methods. Farm Drainage in the United States—History, Status, and Prospects.

[21] van Schilfgaarde J. (1971). Drainage yesterday, today, and tomorrow. Proceedings of the American Society of Agricultural Engineers National Drainage Symposium.

[22] Allen J. (1970). Prehistoric Agricultural Systems in the Waghi Valley—A further note. Mankind.

[23] Ballard C. (2001). Wetland drainage and agricultural transformations in the Southern Highlands of Papua New Guinea. Asia Pac. Viewpoint.

[24] Muke J., Mandui H. (2003). In the shadows of Kuk: Evidence for prehistoric agriculture at Kana, Wahgi Valley, Papua New Guinea. Archaeol. Oceania.

[25] Denham T. (2003). Archaeological evidence for mid-Holocene agriculture in the interior of Papua New Guinea: A critical review. Archaeol. Oceania.

[26] Pavelis G.A. (1987). Farm Drainage in the United States: History, Status, and Prospects 1987.

[27] Blomqvist M.M., Vos P., Klinkhamer G.L., ter Keurs W.J. (2003). Declining plant species richness of grassland ditch banks—A problem of colonisation or extinction?. Biol. Conser..

[28] Hietala-Koivu R., Lankoski J., Tarmi S. (2004). Loss of biodiversity and its social cost in an agricultural landscape. Agri. Ecosyst. Environ..

[29] Helm A., Hanski I., Portel M. (2006). Slow response of plant species richness to habitat loss and fragmentation. Ecol. Lett..

[30] Herzon I., Helenius J. (2008). Agricultural drainage ditches, their biological importance and functioning. Biol. Conser..

[31] USEPA National Water Quality Inventory: Report to Congress, 2004 Reporting Cycle (EPA 841-R-08–001) 2009. http://water.epa.gov/lawsregs/guidance/cwa/305b/2004report_index.cfm.

[32] USEPA National Assessment Database. http://iaspub.epa.gov/waters10/w305b_report_v2.nation.

[33] de Wit M., Behrendt H., Bendoricchio G., Bleuten W., van Gaans P. (2002). The Contribution of Agriculture to Nutrient Pollution in Three European Rivers, with Reference to the European Nitrates Directive; European Water Management Online.

[34] Mourad D.S.J., Van Der Perk M., Piirimäe K. (2006). Changes in nutrient emissions, fluxes and retention in a north-eastern European lowland drainage basin. Environ. Monit. Assess..

[35] Ongley E.D., Xiaolan Z., Tao Y. (2010). Current status of agricultural and rural non-point source pollution assessment in China. Environ. Pollut..

[36] Qu H.J., Kroeze C. (2012). Nutrient export by rivers to the coastal waters of China: management strategies and future trends. Reg. Environ. Change.

[37] Khaleel R., Reddy K.R., Overcash M.R. (1980). Transport of potential pollutants in runoff water from land areas receiving animal wastes: a review. Water Res..

[38] Smukler S.M., O'Geen A.T., Jackson L.E. (2012). Assessment of best management practices for nutrient cycling: a case study on an organic farm in Mediterranean-type climate. J. Soil Water Conserv..

[39] Kideys A.E. (2002). Fall and rise of the Black Sea ecosystem. Science.

[40] Hefner J.M., Brown J.D. (1985). Wetland trends in the southeastern United States. Wetlands.

[41] Dahl T.E. (1990). Wetland losses in the United States, 1780’s to 1980’s.

[42] Foote A.L., Pandey S., Krogman N.T. (1996). Processes of wetland loss in India. Environ. Conserv..

[43] Davis J.A., Froend R. (1999). Loss and degradation of wetlands in southwestern Australia: underlying causes, consequences and solutions. Wetl. Ecol. Manag..

[44] Coleman J.M., Huh O.K., Braud D. (2008). Wetland loss in world deltas. J. Coastal Res..

[45] Zhang J., Ma K., Fu B. (2010). Wetland loss under the impact of agricultural development in the Sanjiang Plain, NE China. Environ. Monit. Assess..

[46] Rudis V.A. (1995). Regional forest fragmentation effects on bottomland hardwood community types and resource values. Landscape Ecol..

[47] Twedt D.J., Loesch C.R. (1999). Forest area and the distribution in the Mississippi Alluvial Valley: Implications for breeding bird conservation. J. Biogeogr..

[48] MacDonald P.O., Frayer W.E., Clauser J.K. (1979). Documentation, chronology, and future projections of bottomland hardwood habitat loss in the Lower Mississippi Alluvial Plain, Volume 1. Technical Report for U.S. Department of the Interior.

[49] Brown R.G. (1998). Effects of wetland channelization on runoff and loading. Wetlands.

[50] Hey D.L., Philippi N.S. (1995). Flood reduction through wetland restoration: the Upper Mississippi River Basin as a case history. Restor. Ecol..

[51] Shankman D., Pugh T.B. (1992). Discharge response to channelization of a coastal plain stream. Wetlands.

[52] Criss R.E., Shock E.L. (2001). Flood enhancement through flood control. Geology.

[53] Steiger J., Tabacchi E., Dufour S., Corenblit D., Peiry J.L. (2005). Hydrogeomorphic processes affecting riparian habitat within alluvial channel-floodplain river systems: a review for the temperate zone. River Res. Appl..

[54] Sophocleous M. (2002). Interactions between groundwater and surface water: the state of the science. Hydrogeol. J..

[55] Skaggs R.W., Chescheir G.M., Phillips B.D. (2005). Methods to determine lateral effect of a drainage ditch on wetland hydrology. Trans. ASAE.

[56] Hill A.R. (1976). The environmental impacts of agricultural land drainage. J. Environ. Manage..

[57] Schlosser I.J., Karr J.R. (1981). Riparian vegetation and channel morphology impact on spatial patterns of water quality in agricultural watersheds. Environ. Manage..

[58] Peterjohn W.T., Correll D.L. (1984). Nutrient dynamics in an agricultural watershed: observations on the role of a riparian forest. Ecology.

[59] Skaggs R.W., Breve M.A., Gilliam J.W. (1994). Hydrologic and water quality impacts of agricultural drainage. Critical Rev. Environ. Sci. Technol..

[60] Thomas D.L., Perry C.D., Evans R.O., Izuno F.T., Stone K.C., Gilliam J.W. (1995). Agricultural drainage effects on water quality in Southeastern U.S. J. Irrig. Drain. E.-ASCE.

[61] Mainstone C.P., Schofield K. (1996). Agricultural management for nonpoint pollution control, with particular reference to the UK. Eur. Water Pollut. Contr..

[62] Magner J., Steffen L. Stream morphological response to climate and land-use in the Minnesota River Basin. Proceedings of the American Society of Civil. Engineers Joint Water Resources Engineering, Planning and Management Conference.

[63] Shields F.D., Knight S.S., Cooper C.M. (1994). Effects of channel incision on base flow stream habitats and fishes. Environ. Manage..

[64] Shields F.D., Knight S.S., Cooper C.M. (1998). Rehabilitation of aquatic habitats in warmwater streams damaged by channel incision in Mississippi. Hydrobiologia..

[65] Hrody P.J., Sutton T.M. (2008). Fish community responses to half-log additions in warmwater streams. N. Am. J. Fish. Manage..

[66] Smiley P.C., Gillespie R.B., Moore M.T., Kröger R. (2010). Influence of physical habitat and agricultural contaminants on fishes within agricultural drainage ditches. Agricultural Drainage Ditches: Mitigation Wetlands for the 21^st^ Century.

[67] McRae S.E., Allan J.D., Burch J.D. (2004). Reach- and catchment-scale determinants of the distribution of freshwater mussels (Bivalvia: Unionidae) in south-eastern Michigan, USA. Freshwater Biol..

[68] Pool K.E., Downing J.A. (2004). Relationship of declining mussel biodiversity to stream-reach and watershed characteristics in an agricultural landscape. J. N. Am. Benthol. Soc..

[69] Downing J.A., Van Meter P., Woolnough D.A. (2010). Suspects and evidence: A review of the causes of extirpation and decline in freshwater mussels. Anim. Biodivers. Conserv..

[70] Robinson M., Rycroft D.W., Skaggs R.W., van Schilfgaarde J. (1999). Chapter 23: The impact of drainage on streamflow. Agricultural Drainage.

[71] Knox J.C. (2001). Agricultural influence on landscape sensitivity in the Upper Mississippi River Valley. Catena.

[72] Knox J.C. (2006). Floodplain sedimentation in the Upper Mississippi Valley: Natural *versus* human accelerated. Geomorphology.

[73] Poff N.L., Allan J.D., Bain M.B., Karr J.R., Prestegaard K.L., Richter B.D., Sparks R.E., Stromberg J.C. (1997). The natural flow regime. BioScience.

[74] Zaimes G.N., Schultz R.C., Isenhart T.M. (2004). Stream bank erosion adjacent to riparian forest buffers, row-crop fields, and continuously-grazed pastures along Bear Creek in central Iowa. J. Soil Water Conserv..

[75] Magner J.A., Payne G.A., Steffen L.J. (2004). Drainage effects on stream nitrate-N and hydrology in south-central Minnesota (USA). Environ. Monit. Assess..

[76] King K.W., Smiley P.C., Fausey N.R. (2009). Hydrology of channelized and natural headwater streams. Hydrol. Sci. J..

[77] Simon A. (1989). The discharge of sediment in channelized alluvial streams. J. Am. Water Resour. A.

[78] Bengtson R.L., Carter C.E., Morris H.F., Bartkiewicz S.A. (1988). Nitrogen and phosphorus losses under subsurface drainage practices in southern Louisiana. Proc. ASAE.

[79] Woltemade C.J. (2000). Ability of restored wetlands to restore nitrogen and phosphorous concentrations in agricultural drainage water. J. Soil Water Conserv..

[80] Sims J.T., Simard R.R., Joern B.C. (1998). Phosphorus losses in agricultural drainage: historical perspective and current research. J. Environ. Qual..

[81] Gentry L.E., David M.B., Royer T.V., Mitchell C.A., Starks K.M. (2007). Phosphorus transport pathways to streams in tile-drained agricultural watersheds. J. Environ. Qual..

[82] Zucker L.A., Brown L.C., The Ohio State University Extension (1988). Agricultural drainage: Water quality impacts and subsurface drainage studies in the Midwest. Ohio State University Extension Bulletin 871.

[83] Sugg Z. (2007). Assessing U.S. Farm Drainage: Can GIS Lead to Better Estimates of Subsurface Drainage Extent?.

[84] Vought L.B.-M., Lacoursière J.O., Eiseltová M. (2010). Restoration of streams in the agricultural landscape. Restoration of Lakes, Streams, Floodplains, and Bogs in Europe; Principles and Case Studies.

[85] Siebert S., Burke J., Faures J.M., Frenken K., Hoogeveen J., Döll P., Portmann F.T. (2010). Groundwater use for irrigation—A global inventory. Hydrol. Earth Syst. Sci. Discuss..

[86] Wen F., Chen X. (2006). Evaluation of the impact of groundwater irrigation on streamflow in Nebraska. J. Hydrol..

[87] Rugel K., Jackson C.R., Romeis J.J., Golladay S.W., Hicks D.W., Dowd J.F. (2012). Effects of irrigation withdrawals on streamflows in a karst environment: lower Flint River Basin, Georgia, USA. Hydrol. Process..

[88] Horton J.L., Kolb T.E., Hart S.C. (2001). Physiological response to groundwater depth varies among species and with river flow regulation. Eco. Appl..

[89] Vitousek P.M., Moonery H.A., Lubchencho J., Melillo J.M. (1997). Human domination of Earth’s ecosystems. Science.

[90] Caraco N.F., Cole J.J. (1999). Human impact on nitrate export: an analysis using major world rivers. AMBIO..

[91] Birgand F., Skaggs R.W., Chescher G.M., Gilliam J.W. (2007). Nitrogen removal in streams in agricultural catchments—A literature review. Critical Reviews in Environ. Sci. Technol..

[92] Turner R.E., Rabalais N.N. (1991). Changes in Mississippi River water quality this century. BioScience.

[93] Shields F.D., Lizotte R.E., Knight S.S., Cooper C.M., Wilcox D. (2010). The stream channel incision syndrome and water quality. Eco. Eng..

[94] Howarth R.W., Jensen H.S., Marino R., Postma H., Tiessen H. (1995). Transport to and processing of P in near-shore and oceanic waters. Phosphorus in the Global Environment: Transfers, Cycles, and Management.

[95] Schilling K.E., Li Z., Zhang Y. (2006). Groundwater-surface water interaction in the riparian zone of an incised channel, Walnut Creek, Iowa. J. Hydrol..

[96] Camargo J.A., Alonso A., Salamanca A. (2005). Nitrate toxicity to aquatic animals: A review with new data for freshwater invertebrates. Chemosphere.

[97] Haywood G.P. (1983). Ammonia toxicity in teleost fish: A review.

[98] Arthur J.W., West C.W., Allen K.N., Hedtke S.F. (1987). Seasonal toxicity of ammonia to five fish and nine invertebrate species. B. Environ. Contam. Tox..

[99] Jofre M.B., Karasov W.H. (1999). Direct effect of ammonia on three species of North American anuran amphibians. Environ. Toxicol. Chem..

[100] Ortiz M.E., Marco A., Saiz N., Lizana M. (2004). Impact of ammonium nitrate on growth and survival of six European amphibians. Arch. of Environ. Contam. and Toxicol..

[101] Smith G.R., Temple K.G., Vaala D.A., Dingfelder H.A. (2005). Effects of nitrate on the tadpoles of two ranids (*Rana. catesbeiana* and *R. clamitans*). Arch. Env. Contam. Toxicol..

[102] Britto D.T., Kronzucker H.J. (2002). NH_4_^+^ toxicity in higher plants: A critical review. J. Plant. Physiol..

[103] USEPA Draft 2009 update. Aquatic Life ambient water quality criteria for ammonia—freshwater. EPA-82-D-09–001 2009.

[104] Carpenter S.R., Caraco N.F., Correll D.L., Sharpley A.N., Smith V.H. (1998). Nonpoint pollution of surface waters with phosphorus and nitrogen. Eco. Appl..

[105] Bennett E.M., Carpenter S.R., Caraco N.F. (2001). Human impact on erodible phosphorus and eutrophication: A global perspective. BioScience.

[106] Evans-White M.A., Dodds W.K., Huggins D.G., Baker D.S. (2009). Thresholds in macroinvertebrate biodiversity and stoichiometry across water-quality gradients in Central Plains (USA) streams. J. N. Am. Benthol. Soc..

[107] Jeppesen E., Søndergaard M., Jensen J.P., Havens K.E., Anneville O., Carvalho L., Coveney M.F., Deneke R., Dokulil M.T., Foy B. (2005). Lake responses to reduced nutrient loading—An analysis of contemporary long-term data from 35 case studies. Freshwater Biol..

[108] Thomaz S.M., Dibble E.D., Evangelista L.R., Higuti J., Bini L.M. (2008). Influence of aquatic macrophyte habitat complexity on invertebrate abundance and richness in tropical lagoons. Freshwater Biol..

[109] Portielje R., Roijackers R.M.M. (1995). Primary succession of aquatic macrophytes in experimental ditches in relation to nutrient input. Aquat. Bot..

[110] Newman S., Grace J.B., Koebel J.W. (1996). Effects of nutrients and hydroperiod on *Typha., Cladium.* and *Eleocharis.*: Implications for Everglades restoration. Eco. Appl..

[111] Lorenzen B., Brix H., Mendelssohn I.A., McKee K.L., Miao S.L. (2001). Growth, biomass allocation and nutrient use efficiency in *Cladium. jamaicense* and *Typha. domingensis* as affected by phosphorus and oxygen availability. Aquat. Bot..

[112] Diaz R.J. (2001). Overview of hypoxia around the world. J. Environ. Qual..

[113] Diaz R.J., Rosenberg R. (2008). Spreading dead zones and consequences for marine ecosystems. Science.

[114] Nixon S.W., Oviatt C.A., Frithsen J., Sullivan B. (1986). Nutrients and the productivity of estuarine and coastal marine ecosystems. J. Limnol. Soc. S. Afr..

[115] Micheli F. (1999). Eutrophication, fisheries, and consumer-resource dynamics in marine pelagic ecosystems. Science.

[116] Baird D., Christian R.R., Peterson C.H., Johnson G.A. (2004). Consequences of hypoxia on estuarine ecosystem function: energy diversion from consumers to microbes. Eco. Appl..

[117] Heisler J., Glibert P.M., Burkholder J.M., Anderson D.M., Cochlan W., Dennison W.C., Dortch Q., Gobler C.J., Heil C.A., Humphries E. (2008). Eutrophication and harmful algal blooms: a scientific consensus. Harmful Algae.

[118] Rabalais N.N. (2002). Nitrogen in aquatic ecosystems. AMBIO.

[119] Seitzinger S.P., Kroeze C., Bouwman A.F., Caraco N., Dentener F., Styles R.V. (2002). Global patterns of dissolved inorganic and particulate nitrogen inputs to coastal system: Recent conditions and future projections. Estuaries Coasts.

[120] Bagge O., Nielsen E., Mellergaard S., Dalsgaard I. Hypoxia and the demersal fish stock in the Kattegat (IIIa) and Subdivision 22. Proceedings of ICES Council Meeting 1990.

[121] Mee L.D. (1992). The Black Sea in crisis: A need for concerted international action. AMBIO.

[122] Österblom H., Hansson S., Larsson U., Hjerne O., Wulff F., Elmgren R., Folke C. (2007). Human-induced trophic cascades and ecological regime shifts in the Baltic Sea. Ecosystems.

[123] Chesney E.J., Baltz D.M., Rabalais N.N., Turner R.E. (2001). The effects of hypoxia on the northern Gulf of Mexico coastal ecosystem: A fisheries perspective. Coastal Hypoxia: Consequences for Living Resources and Ecosystems.

[124] Craig J.K., Crowder L.B. (2005). Hypoxia-induced habitat shifts and energetic consequences in Atlantic croaker and brown shrimp on the Gulf of Mexico shelf. Mar. Ecol. Prog. Ser..

[125] O’Connor T., Whitall D. (2007). Linking hypoxia to shrimp catch in the northern Gulf of Mexico. Mar. Pollut. Bull..

[126] (2001). Mississippi River/Gulf of Mexico Watershed Nutrient Task Force. Action Plan for Reducing, Mitigating, and Controlling Hypoxia in the Northern Gulf of Mexico.

[127] USEPA Gulf Hypoxia Action Plan 2008 for reducing, mitigating and controlling hypoxia in the Northern Gulf of Mexico and improving water quality in the Mississippi River Basin. Mississippi River Gulf of Mexico Watershed Nutrient Task Force. http://water.epa.gov/type/watersheds/named/msbasin/upload/2008_8_28_msbasin_ghap2008_update082608.pdf.

[128] GOMA (Gulf of Mexico Alliance) Governors’ Action Plan II: For Healthy and Resilient Coasts. Gulf of Mexico Alliance, 2009. http://gulfofmexicoaliance.org/pdfs/ap2_final2.pdf.

[129] Mitsch W.J., Gosselink J.G. (2000). Wetlands.

[130] Reddy K.R., DeLaune R.D. (2008). Biogeochemistry of Wetlands.

[131] Kadlec R.H., Wallace S.D. (2009). Treatment Wetlands.

[132] Hilderbrand R.H., Watts A.C., Randle A.M. (2005). The myths of restoration ecology. Eco. Soc..

[133] Moreno-Mateos D., Power M.E., Comın F.A., Yockteng R. (2012). Structural and functional loss in restored wetland ecosystems. PLOS Biol..

[134] Faulkner S., Barrow W., Keeland B., Walls S., Telesco D. (2011). Effects of conservation practices on wetland ecosystem services in the Mississippi Alluvial Valley. Eco. Appl..

[135] Maltby E., Acreman M.C. (2011). Ecosystem services of wetlands: Pathfinder for a new paradigm. Hydrol. Sci. J..

[136] Breukelaar A.W., Lammens E.H.R.R., Klein Breteler J.G.P., Tatrai I. (1994). Effects of benthivorous bream (*Abramis. brama*) and carp (*Cyprinus. carpio*) on sediment resuspension and concentrations of nutrients and chlorophyll *a*. Freshwater Biol..

[137] Post D.M., Taylor J.P., Kitchell J.F., Olsen M.H., Schindler D.E., Herwig B.R. (1998). The role of migratory waterfowl as nutrient vectors in a managed wetland. Conserv. Biol..

[138] Kitchell J.F., Schindler D.R., Herwig B.R., Post D.M., Olson M.H. (1999). Nutrient cycling at the landscape scale: The role of diel foraging migrations by geese at the Bosque del Apache National Wildlife Refuge, New Mexico. Limnol. Oceanogr..

[139] Tomer M.D., Locke M.A. (2011). The challenge of documenting water quality benefits of conservation practices: A review of USDA-ARS’s conservation effects assessment project watershed studies. Water Sci. Technol..

[140] Liira J., Schmidt T., Aavik T., Arens P., Augenstein I., Bailey D., Billeter R., Bukacek R., Burel F., De Blust G. (2008). Plant functional group composition and large-scale species richness in European agricultural landscapes. J. Veg. Sci..

[141] Manhoudt A.G.E., Visser A.J., de Snoo G.R. (2007). Management regimes and farming practices enhancing plant species richness on ditch banks. Agri. Ecosyst. Environ..

[142] de Snoo G.R., van der Poll R.J. (1999). Effect of herbicide drift on adjacent boundary vegetation. Agri. Ecosyst. Environ..

[143] TerHaar M.J., Herricks E.E. (1989). Management and development of aquatic habitat in agricultural drainage systems, Technical Report for Water Resources Center.

[144] Smiley P.C., King K.W., Fausey N.R. (2011). Influence of herbaceous riparian buffers on physical habitat, water chemistry, and stream communities within channelized agricultural headwater streams. Eco. Eng..

[145] Tomer M.D., Dosskey M.G., Burkart M.R., James D.E., Helmers M.J., Eisenhauer D.E. (2009). Methods to prioritize placement of riparian buffers for improved water quality. Agroforesty. Systems.

[146] Schultz R.C., Isenhart T.M., Simpkins W.W., Colletti J.P. (2004). Riparian forest buffers in agroecosystems—lessons learned from the Bear Creek Watershed, central Iowa, USA. Agroforest. Syst..

[147] Bentrup G. (2008). Conservation Buffers: Design Guidelines for Buffers, Corridors, and Greenways. Gen. Tech. Rep. SRS-109.

[148] Haycock N.E., Muscutt A.D. (1995). Landscape management strategies for the control of diffuse pollution. Landscape Urban. Plan..

[149] Lyons J., Trimble S.W., Pain L.K. (2000). Grass *versus* trees: managing riparian areas to benefit streams of central North America. J. Am. Water Resour. A.

[150] Parkyn S. (2004). Review of Riparian Buffer Zone Effectiveness. Technical Paper for Ministry of Agriculture and Forestry.

[151] Pankau R.C., Schoonover J.E., Williard K.W.J., Edwards P.J. (2012). Concentrated flow paths in riparian buffer zones of southern Illinois. Agroforestry. Systems.

[152] Vought L.B.-M., Pinay G., Fuglsang A., Ruffioni C. (1995). Structure and function of buffer strips from a water quality perspective in agricultural landscapes. Landscape Urban Plann..

[153] Blackwell M.S.A., Hogan D.V., Pinay G., Maltby E., Maltby E., Barker T. (2009). The role of buffer zones for agricultural runoff. The Wetlands Handbook.

[154] Parkyn S.M., Davies-Colley R.J., Cooper A.B., Stroud M.J. (2005). Predictions of stream nutrient and sediment yield changes following restoration of forested riparian buffers. Eco. Eng..

[155] Sarriquet P.E., Delettre Y.R., Marmonier P. (2006). Effects of catchment disturbance on stream invertebrates: Comparison of different habitats (vegetation, benthic, and interstitial) using bio-ecological groups. Ann. Limnol.-Int. J. Limn..

[156] Haycock N.E., Pinay G. (1993). Groundwater nitrate dynamics in grass and poplar vegetated riparian buffers during the winter. J. Environ. Qual..

[157] Hefting M.M., Clement J.C., Bienkowski P., Dowrick D., Guenat C., Butturini A., Topa S.T., Pinay G., Verhoeven J.T.A. (2005). The role of vegetation and litter in the nitrogen dynamics of riparian buffer zones in Europe. Eco. Eng..

[158] Sweeney B.W., Bott T.L., Jackson J.K., Kaplan L.A., Newbold J.D., Standley L.J., Hession W.C., Horwitz R.J. (2004). Riparian deforestation, stream narrowing, and loss of stream ecosystem services. Proc. Nat. Acad. Sci. USA.

[159] Clément J.C., Pinay G., Marmonier P. (2002). Seasonal Dynamics of Denitrification along Topohydrosequences in Three Different Riparian Wetlands. J. Environ. Qual..

[160] Kuusemets V., Mander U., Lohmus K., Ivask M. (2001). Nitrogen and phosphorus variation in shallow groundwater and assimilation in plants in complex riparian buffer zones. Water Sci. Technol..

[161] Bunn S.E., Davies P.M., Kellaway D.M., Prosser I.P. (1998). Influence of invasive macrophytes on channel morphology and hydrology in an open tropical lowland stream, and potential control by riparian shading. Freshwater Biol..

[162] Paine L.K., Ribic C.A. (2002). Comparison of riparian plant communities under four land management systems in southwestern Wisconsin. Agri. Ecosyst. Environ..

[163] Parkyn S.M., Davies-Colley R.J., Halliday N.J., Costley K.J., Croker G.F. (2003). Planted Riparian Buffer Zones in New Zealand: Do They Live Up to Expectations?. Restor. Ecol..

[164] Dodds W.K., Whiles M.R. (2010). Freshwater Ecology: Concepts & Environmental Applications of Limnology.

[165] Shankman D. (1996). Stream channelization and changing vegetation patterns in the U.S. Coastal Plain. Geogr. Rev..

[166] Smiley P.C., Shields D.F., Knight S.S. (2009). Designing impact assessments for evaluating ecological effects of agricultural conservation practices on streams. J. Am. Water Resour. A..

[167] Zedler J.B. (2003). Wetlands at your service: Reducing impacts of agriculture at the watershed scale. Front. Ecol. Environ..

[168] Kleinman P.J.A., Sharpley A.N., McDowell R.W., Flaten D.N., Buda A.R., Tao L., Bergstrom L., Zhu Q. (2011). Managing agricultural phosphorus for water quality protection: Principles for progress. Plant Soil.

[169] Alexander R.B., Smith R.A., Schwarz G.E. (2000). Effect of stream channel size on the delivery of nitrogen to the Gulf of Mexico. Nature.

[170] Esselman P.C., Infante D.M., Wang L., Wu D., Cooper A.R, Taylor W.W. (2011). An index of cumulative disturbance to river fish habitats of the conterminous United States from landscape anthropogenic activities. Eco. Res..

[171] Vannote R.L., Minshall G.W., Cummins K.W., Sedell J.R., Cushing C.E. (1980). The river continuum concept. Can. J. Fish Aquat. Sci..

[172] Arango C.P., Tank J.L. (2008). Land use influences the spatiotemporal controls on nitrification and denitrification in headwater streams. J. N. Am. Benthol. Soc..

[173] Benke A.C., Henry III R.L., Gillespie D.M., Hunter R.J. (1985). Importance of snag habitat for animal production in southeastern streams. Fisheries.

[174] Julian J.P., Seegert S.Z., Powers S.M., Stanley E.H., Doyle M.W. (2011). Light as a first-order control on ecosystem structure in a temperate stream. Ecohydrology..

[175] Wilcock R.J., Scarsbrook M.R., Costley K.J., Nagels J.W. (2002). Controlled release experiments to determine the effects of shade and plants on nutrient retention in a lowland stream. Hydrobiologia..

[176] Wilcock R.J., Scarsbrook M.R., Cooke J.G., Costley K.J., Nagels J.W. (2004). Shade and flow effects on ammonia retention in macrophyte-rich streams: Implications for water quality. Environ. Pollut..

[177] Collier K.J., Cooper A.B., Davies-Colley R.J., Rutherford J.C., Smith C.M., Williamson R.B. (1995). Managing Riparian Zones: A Contribution to Protecting New Zealand’s Rivers and Streams, Volume 2: Guidelines.

[178] Boutin C., Jobin J., Bélanger L. (2003). Importance of riparian habitats to flora conservation in farming landscape of southern Québec, Canada. Agri. Ecosyst. Environ..

[179] Ryan R.L., Erickson D.L., De Young R. (2003). Farmers' Motivations for Adopting Conservation Practices along Riparian Zones in a Mid-western Agricultural Watershed. J. Environ. Plann. Manage..

[180] Soomers H., Winkel D.N., Wassen Y., Wassen M.J. (2010). The dispersal and deposition of hydrochorous plant seeds in drainage ditches. Freshwater Biol..

[181] Simon T.N., Travis J. (2011). The contribution of man-made ditches to the regional stream biodiversity of the new river watershed in the Florida panhandle. Hydrobiologia..

[182] Shields F.D. Jr., Cooper C.M., Cotroneo G.V., Rumer R.R. (1994). Riparian wetlands and flood stages. Hydraulic Engineering.

[183] Williams D.D., Hynes H.B.N. (1977). The ecology of temporary streams II: General remarks on temporary streams. Int. Rev. Ges. Hydrobio..

[184] Wilde S.A., Steinbrenner E.C., Pierce R.S., Dosen R.C., Pronin D.T. (1953). Influence of forest cover on the state of the ground water table. Soil Sci. Soc. Am. J..

[185] Borg H., Stoneman G.L., Ward C.G. (1987). The effect of logging and regeneration on groundwater, streamflow and stream salinity in the southern forest of Western Australia. J. Hydrol..

[186] Mulholland P.J., Helton A.M., Poole G.C., Hall R.O., Hamilton S.K., Peterson B.J., Tank J.L., Ashkenas L.R., Cooper L.W., Dahm C.N. (2008). Stream denitrification across biomes and its response to anthropogenic nitrate loading. Nature.

[187] Royer T.V., Tank J.L., David M.D. (2004). Transport and fate of nitrate in headwater agricultural streams in Illinois. J. Environ. Qual..

[188] Burt T., Pinay G., Sabater S. (2010). Ecohydrology Bearings—Invited Commentary. What do we still need to know about the ecohydrology of riparian zones?. Ecohydrology..

[189] Samani J.M.V., Kouwen N. (2002). Stability and erosion in grassed channels. J. Hydraul. Eng.-ASCE.

[190] Shields D.F., Smiley P.C. Jr., Cooper C.M., Borselli L. (2007). Modifying erosion control structures for ecological benefits. J. Soil Water Conserv..

[191] Shields F.D., Smiley P.C., Cooper C.M. (2002). Design and management of edge-of-field water control structures for ecological benefits. J. Soil Water Conserv..

[192] Smiley P.C., Knight S.S., Shields F.D., Cooper C.M. (2009). Influence of gully erosion control on amphibian and reptile communities within riparian zones of channelized streams. Ecohydrology.

[193] Manley S.W., Kaminski R.M., Rodrigue P.B., Dewey J.C., Schoenholtz S.H., Gerard P.D., Reinecke K.J. (2009). Soil and nutrient retention in winter-flooded rice fields with implications for watershed management. J. Soil Water Conserv..

[194] Rosgen D.L. (1994). River restoration utilizing natural stability concepts. Land Water.

[195] Ward A., Mecklenburg D., Powell G.E., Brown L., Jayakaran A. Two-Stage Channel Design Procedures. Proceedings of the Self-Sustaining Solutions for Streams, Wetlands, and Watersheds Conference.

[196] Powell G.E., Ward A.D., Mecklenburg D.E., Jayakaran A.D. (2007). Two-stage channel systems: Part 1, a practical approach for sizing agricultural ditches. J. Soil Water Conserv..

[197] Kröger R., Holland M.M., Moore M.T., Cooper C.M. (2007). Plant senescence: a mechanism for nutrient release in temperate agricultural wetlands. Environ. Pollut..

[198] Kröger R., Cooper C.M., Moore M.T. (2008). A preliminary study of alternative controlled drainage strategy in surface drainage ditches: Low grade weirs. Agr. Water Manage..

[199] Powell K.L., Bouchard V. (2010). Is denitrification enhanced by the development of natural fluvial morphology in agricultural headwater ditches?. J. N. Am. Benthol. Soc..

[200] Roley S.S., Tank J.L., Stephen M.L., Johnson L.T., Beaulieu J.J., Witter J.D. (2012). Floodplain restoration enhances denitrification and reach-scale nitrogen removal in an agricultural stream. Eco. Appl..

[201] Roley S.S., Tank J.L., Williams W.L. (2012). Hydrologic connectivity increases denitrification in the hyporheic zone and restored floodplains of an agricultural stream. J. Geophys. Res..

[202] Landwehr K., Rhoads B.L. (2003). Depositional response of a headwater stream to channelization, east central Illinois, USA. River Res. Appl..

[203] D’Ambrosio J.L., Ward A., Witter J.D., Tank J.L. Ecological services of constructed two-stage agricultural ditches. proceedings of 21st Century Watershed Technology Conference and Workshop Improving Water Quality and the Environment.

[204] Kramer G. (2011). Design, Construction, and Assessment of a self-sustaining drainage ditch. Master’s Thesis.

[205] Janssen J.R. (2008). Environmental and Management Influences on Fish and Invertebrate Communities in Agricultural Headwater Systems. Master’s Thesis.

[206] Sharpley A.N., Krogstad T., Kleinman P.J.A., Haggard B.E., Shigaki F., Saporito L. (2007). Managing Natural Processes in drainage ditches for non-point source phosphorus control. J. Soil Water Conserv..

[207] Shields F.D. Jr., Pezeshki S.R., Wilson G.V., Wu W., Dabney S.M. (2008). Rehabilitation of an incised stream with plant materials: the dominance of geomorphic processes. Eco. Soc..

[208] Gilliam J.W., Skaggs R.W. (1986). Controlled agricultural drainage to maintain water quality. J. Irrig. Drain. E. ASCE.

[209] Wesström I., Messing I., Linner H., Lindstrom J. (2001). Controlled drainage—Effects on drain outflow and water quality. Agr. Water Manage..

[210] Simon A., Darby S.E. (2002). Effectiveness of grade-control structures in reducing erosion along incised river channels: the case of Hotophia Creek, Mississippi. Geomorphology.

[211] Needelman B.A., Ruppert D.E., Vaughan R.E. (2007). The role of ditch soil formation and redox biogeochemistry in mitigating nutrient and pollutant losses from agriculture. J. Soil Water Conserv..

[212] Needelman B.A., Kleinman P.J.A., Strock J.S., Allen A.L. (2007). Improved management of agriculture drainage ditches for water quality protection: An overview. J. Soil Water Conserv..

[213] Woli K.P., David M.B., Cooke R.A., McIsaac G.F., Mitchell C.A. (2010). Nitrogen balance in and export from agricultural fields associated with controlled drainage systems and denitrfying bioreactors. Eco. Eng..

[214] Penn C.J., Bryant R.B, Kleinman P.J.A., Allen A.L. (2007). Removing dissolved phosphorus from drainage ditch water with phosphorus sorbing materials. J. Soil Water Conserv..

[215] Penn C.J., McGrath J.M., Bryant R.B., Moore M.T., Kröger R. (2010). Ditch Drainage Management for Water Quality Improvement. Agricultural Drainage Ditches: Mitigation Wetlands for the 21st Century.

[216] Van der Hoven S.J., Fromm N.J., Peterson E.W. (2008). Quantifying nitrogen cycling beneath a meander of a low gradient, N-impacted, agricultural stream using tracers and numerical modeling. Hydrol. Process..

[217] Boulton A.J. (2007). Hyporheic rehabilitation in rivers: Restoring vertical connectivity. Freshwater Biol..

[218] Peterson E.W., Benning C. (2012). Factors influencing nitrate within a low-gradient agricultural stream. Environ. Earth Sci..

[219] Wondzell S.M., LaNier J., Haggerty R., Woodsmith R.D., Edwards R.T. (2009). Changes in hyporheic exchange flow following experimental wood removal in a small, low-gradient stream. Water Resour. Res..

[220] Grimaldi C., Chaplot V. (2000). Nitrate depletion during within-stream transport: Effects of exchange processes between streamwater, the hyporheic and riparian zones. Water Air Soil Poll..

[221] Lefebvre S., Marmonier P., Bour O., Aqulina L., Baudy J. (2005). Nutrient dynamics in interstitial low-order rural streams with different bedrock geology. Arch. Hydrol..

[222] Kasahara T., Hill A.R. (2006). Effects of riffle–step restoration on hyporheic zone chemistry in N-rich lowland streams. Can. J. Fish. Aquat. Sci..

[223] Kasahara T., Hill A.R. (2007). Instream restoration: its effects on lateral stream-subsurface water exchange in urban and agricultural streams in southern Ontario. River Res. Appl..

[224] Sawyer A.H., Cardenas M.B., Buttles J. (2011). Hyporheic exchange due to channel-spanning logs. Water Resour. Res..

[225] Borin M., Bonaiti G., Giardini L. (2001). Controlled drainage and wetlands to reduce agricultural pollution: A lysimetric study. J. Environ. Qual..

[226] Fausey N.R. (2005). Drainage management for humid regions. Int. Agr. Eng. J..

[227] Groffman P.M., Dorsey A.M., Mayer P.M. (2005). N processing within geomorphic structures in urban streams. J. N. Am. Benthol. Soc..

[228] Filoso S., Palmer M.A. (2011). Assessing stream restoration effectiveness at reducing nitrogen export to downstream waters. Eco. Appl..

[229] Lautz L.K., Fanelli R.M. (2008). Seasonal biogeochemical hotspots in the streambed around restoration structures. Biogeochemistry.

[230] Robertson W.D., Merkley L.C. (2009). In-stream bioreactor for agricultural nitrate treatment. J. Environ. Qual..

[231] Lalonde V., Madramootoo C.A., Trenhold L., Broughton R.S. (1996). Effects of controlled drainage on nitrate concentrations in subsurface drain discharge. Agr. Water Manage..

[232] Gilliam J.W., Skaggs R.W., Weed S.B. (1979). Drainage control to diminish nitrate loss from agricultural fields. J. Environ. Qual..

[233] Evans R.O., Gilliam J.W., Skaggs R.W. (1991). Controlled Drainage Management Guidelines For Improving Water Quality.

[234] Evans R.O., Skaggs R.W., Gilliam J.W. (1995). Controlled *versus* conventional drainage effects on water quality. J. Irrig. Drain. E. ASCE.

[235] Kröger R., Moore M.T., Jerry L., Farris J.L., Gopalan M. (2011). Evidence for the Use of Low-Grade Weirs in Drainage Ditches to Improve Nutrient Reductions from Agriculture. Water Air Soil Poll..

[236] Kröger R., Pierce S.C., Littlejohn K.A., Moore M.T., Farris J.L. (2012). Decreasing nitrate-N loads to coastal ecosystems with innovative drainage management strategies in agricultural landscapes: An experimental approach. Agr. Water Manage..

[237] Wesström I., Messing I. (2007). Effects of controlled drainage on N and P losses and N dynamics in a loamy sand with spring crops. Agr. Water Manage..

[238] Ng H.Y.F., Tan C.S., Drury C.F., Gaynor J.D. (2001). Controlled drainage and subirrigation influences tile nitrate loss and corn yields in a sandy loam soil in Southwestern Ontario. Agri. Ecosyst. Environ..

[239] Tan C.S., Drury C.F., Soultani M., vanWesenbeeck I.J., Ng H.Y.F., Gaynor J.D., Welacky T.W. (1998). Effect of controlled drainage and tillage on soil structure and tile drainage nitrate loss at the field scale. Water Sci. Technol..

[240] Drury C.F., Tan C.S., Reynolds W.D., Welacky T.W., Oloya T.O., Gaynor J.D. (2009). Managing Tile Drainage, Subirrigation, and Nitrogen Fertilization to Enhance Crop Yields and Reduce Nitrate Loss. J. Environ. Qual..

[241] Bastienė N., Šaulienė The impact of controlled drainage on water quality. Research for Rural Development 2009. Proceedings of Annual 15th International Scientific Conference.

[242] Pierce S.C., Kröger R. (2011). Low-grade weirs in agricultural ditches for sediment retention and nutrient reduction create in-stream wetlands. Wetland Sci. Pract..

[243] Blackwell M.S.A., Pilgrim E.S. (2011). Ecosystem services delivered by small-scale wetlands. Hydrol. Sci. J..

[244] Hunt P.G., Stone K.C., Humenik F.J., Matheny T.A., Johnson M.H. (1999). In-stream wetland mitigation of nitrogen contamination in a USA coastal plain stream. J. Environ. Qual..

[245] O’Geen A.T., Maynard J.J., Dahlgren R.A. (2007). Efficacy of constructed wetlands to mitigate non-point source pollution from irrigation tailwaters in the San Joaquin Valley, California, USA. Water Sci. Technol..

[246] Tanner C.C., Nguyen M.L., Sukias J.P.S. (2005). Nutrient removal by a constructed wetland treating subsurface drainage from grazed dairy pasture. Agri. Ecosyst. Environ..

[247] Sukias J., Tanner C., Currie L.D., Christensen C.L. (2011). Surface flow constructed wetland as a drainage management tool—long term performance. Adding to the Knowledge Base for the Nutrient Manager, Fertilizer & Lime Research Centre, Occasional Report No. 24.

[248] Koskiaho J., Ekholm P., Räty M., Riihimäki J., Puustinen M. (2003). Retaining agricultural nutrients in constructed wetlands—Experiences under boreal conditions. Eco. Eng..

[249] Kovacic D.A., Twait R.M., Wallace M.P., Bowling J.M. (2006). Use of created wetlands to improve water quality in the Midwest—Lake Bloomington case study. Eco. Eng..

[250] Braskerud B.C. (2002). Factors affecting nitrogen retention in small constructed wetlands treating agricultural non-point source pollution. Eco. Eng..

[251] Braskerud B.C. (2002). Factors affecting phosphorus retention in small constructed wetlands treating agricultural non-point source pollution. Eco. Eng..

[252] Tomer M., Tanner C., Howard-Williams C. (2009). Discussing wetlands, agriculture, and ecosystem services. Wetland Sci. Pract..

[253] Pierce S.C., Kröger R., Prevost D., Poganski B., Flora C., Pierce T. Field-scale monitoring of agricultural ditches as conduits of nitrogen, phosphorus, and suspended sediment in response to storm events and low-input drainage management: A case-study of the Tchula Lake Farm. Proceedings of the Mississippi Water Resources Conference.

[254] Shields F.D., Knight S.S., Cooper C.M. (2007). Can warmwater streams be rehabilitated using watershed-scale standard erosion control measures alone?. Environ. Manage..

[255] Litvan M.E., Stewart T.W., Pierce C.L., Larson C.J. (2008). Effects of grade control structures on the macroinvertebrate assemblage of an agriculturally-impacted stream. River Res. Appl..

[256] Litvan M.E., Stewart T.W., Pierce C.L., Larson C.J. (2008). Fish Assemblages in a Western Iowa Stream Modified by Grade Control Structures. N. Am. J. Fish. Manage..

[257] Santucci V.J., Gephard S.R., Pescitelli S.M. (2005). Effects of Multiple Low-Head Dams on Fish, Macroinvertebrates, Habitat, and Water Quality in the Fox River, Illinois. N. Am. J. Fish. Manage..

[258] Swift M.J., Izac A.M.N., van Noordwijk M. (2004). Biodiversity and ecosystem services in agricultural landscapes—Are we asking the right questions?. Agri. Ecosys. Environ..

[259] MacArthur R.H. (1955). Fluctuations of animal populations and a measure of community stability. Ecology.

[260] Griffin J.N., O’Gorman E.J., Emmerson M.C., Jenkins S.R., Klein A.M., Loreau M., Symstad A., Naeem S., Bunker D.E., Hector A., Loreau M., Perrings C. (2009). Biodiversity and the stability of ecosystem functioning. Biodiversity, Ecosystem Functioning, and Human Wellbeing—An Ecological and Economic Perspective.

[261] Doak D.F., Bigger D., Harding E.K., Marvier M.A., O’Malley R.E., Thomson D. (1998). The statistical inevitability of stability–diversity relationships in community ecology. Am. Nat..

[262] Vandewalle M., de Bello F., Berg M.P., Bolger T., Dolédec S., Dubs F., Feld C.K., Harrington R., Harrison P.A., Lavorel S. (2010). Functional traits as indicators of biodiversity response to land use changes across ecosystems and organisms. Biodivers. Conserv..

[263] MacArthur R.H., MacArthur J.W. (1961). On bird species diversity. Ecology.

[264] Armitage P.D., Szoszkiewicz K., Blackburn J.H., Nesbitt I. (2003). Ditch communities: A major contributor to floodplain biodiversity. Aqu. Conserv. Mar. Freshwater Ecosyst..

[265] Hanski I. (1998). Metapopulation Ecology.

[266] Knapp C.W., Dodds W.K., Wilson K.C., O’Brien J.M., Graham D.W. (2009). Spatial heterogeneity of denitrification genes in a highly homogenous urban stream. Environ. Sci. Technol..

[267] Palmer M.A., Menninger H.L., Bernhardt E. (2010). River restoration, habitat heterogeneity and biodiversity: a failure of theory or practice?. Freshwater Biol..

[268] Dimitrakopoulous P.G., Schmid B. (2004). Biodiversity effects increase linearly with biotope space. Eco. Lett..

[269] Pedersen T.C.M., Baattrup-Pedersen A., Madsen T.V. (2006). Effects of stream restoration and management on plant communities in lowland streams. Freshwater Biol..

[270] Vivian-Smith G. (1997). Microtopographic heterogeneity and floristic diversity in experimental wetland communities. J. Ecol..

[271] Lundholm J.T. (2009). Plant Species diversity and environmental heterogeneity: Spatial scale and competing hypotheses. J. Veg. Sci..

[272] Pezeshki S.R. (2001). Wetland plant responses to soil flooding. Environ. Exp. Bot..

[273] Miller R.C., Zedler J.B. (2003). Responses of native and invasive wetland plants to hydroperiod and water depth. Plant Ecol..

[274] Fraser L.H., Karnezis J.P. (2005). A comparative assessment of seedling survival and biomass accumulation for fourteen wetland plant species grown under minor water-depth differences. Wetlands.

[275] Franklin P., Dunbar M., Whitehead P. (2008). Flow controls on lowland river macrophytes: A review. Scie. Total Environ..

[276] Bornette G., Puijalon S. (2011). Response of aquatic plants to abiotic factors: A review. Aquat. Sci..

[277] Lorenz A.W., Korte T., Sundermann A., Januschke K., Haase P. (2012). Macrophytes respond to reach-scale river restorations. J. Appl. Ecol..

[278] Davies B., Biggs J., Williams P., Whitfield M., Nicolet P., Sear D., Bray S., Maund S. (2008). Comparative biodiversity of aquatic habitats in the European agricultural landscape. Agri. Ecosysts. Environ..

[279] Davies B.R., Biggs J., Williams P.J., Lee J.T., Thompson S. (2008). A comparison of the catchment sizes of rivers, streams, ponds, ditches and lakes: implications for protecting aquatic biodiversity in an agricultural landscape. Hydrobiologia..

[280] de Snoo G.R., Naus N., Verhulst J., van Ruijven J., Schaffers A.P. (2012). Long-term changes in plant diversity of grasslands under agricultural and conservation management. Appl. Veg. Sci..

[281] Leng X., Musters C.J.M., de Snoo G.R. (2009). Restoration of plant diversity on ditch banks: Seed and site limitation in response to agri-environment schemes. Biol. Conserv..

[282] Geertsema W., Opdam P., Kropff M.J. (2002). Plant strategies and agricultural landscapes: Survival in spatially and temporally fragmented habitat. Landscape Ecol..

[283] Milsom T.P., Sherwood A.J., Rose S.C., Town S.J., Runham S.R. (2004). Dynamics and management of plant communities in ditches bordering arable fenland in eastern England. Agri. Ecosys. Environ..

[284] Biggs J., Williams P., Whitfield M., Nicolet P., Brown C., Hollis J., Arnold D., Pepper T. (2007). The freshwater biota of British agricultural landscapes and their sensitivity to pesticides. Agri. Ecosyst. Environ..

[285] Beltman B. (1987). Effects of weed control on species composition of aquatic plants and bank plants and macrofauna in ditches. Hydrol. Bull..

[286] Best E.P.H. (1993). The impact of mechanical harvesting regimes on the species composition of Dutch ditch vegetation: A quantitative approach. J. Aquat. Plant Manage..

[287] Blomqvist M.M., Tamis W.L.M., Bakker J.P., Van der Meijden E. (2006). Seed and (micro) site limitation in ditch banks: Germination, establishment and survival under different management regimes. J. Nat. Conserv..

[288] Geertsema W., Sprangers J.T.C.M. (2002). Plant distribution patterns related to species characteristics and spatial and temporal habitat heterogeneity in a network of ditch banks. Plant Ecol..

[289] van Zuidam J.P., Raaphorst E.P., Peeters E.T.H.M. (2012). The role of propagule banks from drainage ditches dominated by free-floating or submerged plants in vegetation restoration. Restor. Ecol..

[290] Mountford J.O. (2006). The vegetation of artificial drainage channels within grazing marshes in the UK: How does its composition correspond with described communities?. Biol. Environ..

[291] Leng X., Musters C.J.M., de Snoo G.R. (2010). Spatial variation in ditch bank plant species composition at the regional level: the role of environment and dispersal. J. Veg. Sci..

[292] Lenssen J., Menting F., van der Putten W., Blom K. (1999). Control of plant species richness and zonation of functional groups along a freshwater flooding gradient. OIKOS.

[293] Silvertown J., Dodd M.E., Gowing D.J.G., Mountford J.O. (1999). Hydrologically defined niches reveal a basis for species richness in plant communities. Nature.

[294] Casanova M.T., Brock M.A. (2000). How do depth, duration and frequency of flooding influence the establishment of wetland plant communities. Plant Ecol..

[295] Best E.P.H., van der Schaaf S., Oomes M.J.M. (1995). Responses of restored grassland ditch vegetation to hydrological changes, 1989–1992. Plant Ecol..

[296] Pezeshki S.R., Anderson P.H., Shields F.D. (1998). Effects of soil moisture regimes on growth and survival of black willow (*Salix nigra*) posts (cuttings). Wetlands.

[297] Li S., Pezeshki S.R., Goodwin S. (2004). Effects of soil moisture regimes on photosynthesis and growth in cattail (*Typha. latifolia*). Acta Oecol..

[298] Pezeshki S.R., Shields F.D. (2006). Black willow cutting survival in streambank plantings, southeastern United States. J. Am. Water Resour. A..

[299] Twisk W., Noordervliet M.A.W., ter Keurs W.J. (2003). The nature value of the ditch vegetation in peat areas in relation to farm management. Aquat. Ecol..

[300] Madsen R.V., Chambers P.A., James W.F., Koch E.W., Westlake D.F. (2001). The interaction between water movement, sediment dynamics and submersed macrophytes. Hydrobiologia..

[301] Schaller J.L., Royer T.V., David M.B., Tank J.L. (2004). Denitrification associated with plants and sediments in an agricultural stream. J. N. Am. Benthol. Soc..

[302] Janse J.H., van Puijenbroek P.J.T.M. (1998). Effects of eutrophication in drainage ditches. Environ. Pollut..

[303] Kočić A., Hengl T., Horvatić J. (2008). Water nutrient concentrations in channels in relation to occurrence of aquatic plants: a case study in eastern Croatia. Hydrobiologia.

[304] Goulder R. (2008). Conservation of aquatic plants in artificial watercourses: are main drains a substitute for vulnerable navigation canals?. Aqu. Conserv. Mar. Freshwater Ecosyst..

[305] Syzmura M., Syzmura T., Dunajski A., Wolski K. (2009). Grasses (*Poaceae.*) in riparian vegetation of watercourses in agriculture landscape. Pol. J. Environ. Stud..

[306] Lu T., Keming M.A., Bojie F.U., Jieyu Z., Lu Q., Hudson S. (2009). Diversity and composition of wetland communities along an agricultural drainage ditch density gradient. Polish J. Ecol..

[307] Pywell R.F., Bullock J.M., Roy D.B., Warman L., Walker K.J., Rothery P. (2003). Plant traits as predictors of performance in ecological restoration. J. Appl. Ecol..

[308] Boutin C., Keddy P.A. (1993). A functional classification of wetland plants. J. Veg. Sci..

[309] Ervin G.N. (2005). Spatio-temporally variable effects of a dominant macrophyte on vascular plant neighbors. Wetlands.

[310] Pierce S.C., Pezeshki S.R., Moore M.T., Kröger R. (2010). Vegetation in agricultural ditches: limitations to establishment, productivity, and ecosystem functioning. Agricultural Drainage Ditches: Mitigation Wetlands of the 21st Century.

[311] Shupryt M.P., Stelzer R.S. (2009). Macrophyte beds contribute disproportionately to benthic invertebrate abundance and biomass in a sand plains stream. Hydrobiologia..

[312] Pederson M.L., Friberg N. (2009). Influence of disturbance on habitats and biological communities in lowland streams. Fundam. Appl. Limnol..

[313] Brix H. (1997). Do macrophytes play a role in constructed treatment wetlands?. Water Sci. Technol..

[314] Toet S., Huibers L.H.F.A., Logtestijn R.S.P.V.L., Verhoeven J.T.A. (2003). Denitrification in the periphyton associated with plant shoots and in the sediment of a wetland system supplied with sewage treatment plant effluent. Hydrobiologia.

[315] Wu Q.T., Gao T., Zeng S., Chua H. (2006). Plant biofilm oxidation ditch for *in situ* treatment of polluted waters. Eco. Eng..

[316] Thomaz S.M., da Cunha E.R. (2010). The role of macrophytes in habitat structuring in aquatic ecosystems: methods of measurement, causes and consequences on animal assemblages’ composition and biodiversity. Acta. Limnol. Bras..

[317] Dibble E.D. (2009). Use of fractal dimension to assess habitat complexity and its influence on dominant invertebrates inhabiting tropical and temperate macrophytes. J. Freshwater Ecol..

[318] Weisner S.E.B., Thiere G. (2010). Effects of vegetation state on biodiversity and nitrogen retention in created wetlands: A test of the biodiversity–ecosystem functioning hypothesis. Freshwater Biol..

[319] Read J., Wevill T., Fletcher T., Deletic A. (2008). Variation among plant species in pollutant removal from stormwater in biofiltration systems. Water Res..

[320] Srivastava J., Gupta A., Chandra H. (2008). Managing water quality with aquatic macrophytes. Rev. Environ. Sci. Biotechnol..

[321] Brisson J., Chazarenc F. (2009). Maximizing pollutant removal in constructed wetlands: Should we pay more attention to macrophyte species selection?. Sci. Total Environ..

[322] Vymazal J. (2011). Plants used in constructed wetlands with horizontal subsurface flow: A review. Hydrobiologia.

[323] Sand-Jensen K. (1998). Influence of submerged macrophytes on sediment composition and near-bed flow in lowland streams. Freshwater Biol..

[324] Kröger R., Moore M.T., Locke M.A., Cullum R.F., Steinriede R.W., Testa S.,  Bryant C.T., Cooper C.M. (2009). Evaluating the influence of wetland vegetation on chemical residence time in Mississippi Delta drainage ditches. Agr. Water Manage..

[325] Asaeda T., Rajapakse L., Kanoh M. (2010). Fine sediment retention as affected by annual shoot collapse: *Sparganium. erectum* as an ecosystem engineer in a lowland stream. River Res. Appl..

[326] Braskerud B.C. (2001). The influence of vegetation on sedimentation and resuspension of soil particles in small constructed wetlands. J. Environ. Qual..

[327] Stringfellow W., Graham J., Rogers M., Borglin S., Brunell M., Hanlon J., Spier C., Nguyen K., Moore M.T., Kröger R. (2010). Water quality changes occurring in agricultural drains of varying riparian function. Agricultural Drainage Ditches: Mitigation Wetlands for the 21st Century.

[328] Horppila J., Nurminen L. (2001). The effect of an emergent macrophyte (*Typha. augustifolia*) on sediment resuspension in a shallow north temperate lake. Freshwater Biol..

[329] Shields F.D. Jr., Bowie A.J., Cooper C.M. (1995). Control of streambank erosion due to bed degradation with vegetation and structure. Water Resour. Bull..

[330] Blom C.W.P.M. (1999). Adaptations to flooding stress: From plant community to molecule. Plant Biol..

[331] Moore M.T., Kröger R., Locke M.A., Cullum R.F., Steinriede R.W., Testa S., Lizotte R.E., Bryant C.T., Cooper C.M. (2010). Nutrient mitigation capacity in Mississippi Delta, USA drainage ditches. Environ. Pollut..

[332] Jiang C., Fan X., Cui G., Zhang Y. (2007). Removal of agricultural non-point source pollutants by ditch wetlands: implications for lake eutrophication control. Hydrobiologia.

[333] Güsewell S., Koerselman W. (2002). Variation in nitrogen and phosphorus concentrations of wetland plants. Perspect. Plant. Ecol..

[334] Shields F.D., Cooper C.M., Testa S., Ursic M.E. (2008). Nutrient Transport in the Yazoo River Basin.

[335] DeBusk T.A., Peterson J.E., Reddy K.R., Graetz D.A., Clough K.S. (1989). Optimization of the vegetative uptake of phosphorus from dairy wastewater.

[336] Barko J.W., Gunnison D., Carpenter S.R. (1991). Sediment interactions with submersed macrophyte growth and community dynamics. Aquat. Bot..

[337] Chen R.L., Barko J.W. (1988). Effects of freshwater macrophytes on sediment chemistry. J. Freshwater Ecol..

[338] Jeperson D.N., Sorrell B.K., Brix H. (1998). Growth and root oxygen release by *Typha latifolia* and its effects on sediment methanogenesis. Aquat. Bot..

[339] Neuman G., Römhel V., Waisel Y., Eshel A., Kafkafi U. (2002). Root-induced changes in the availability of nutrients in the rhizosphere. Plant Roots: The Hidden Half.

[340] Ehrenfeld J.G., Ravit B., Elgersma K. (2005). Feedback in the plant-soil system. Annu. Rev. Environ. Resour..

[341] Rubio G., Oesterheld M., Alvarez C.R., Lavado R.S. (1997). Mechanisms for the increase in phosphorus uptake of water-logged plants: Soil phosphorus availability, root morphology and uptake kinetics. Oecologia.

[342] Pierce S.C., Moore M.T., Larsen D., Pezeshki S.R. (2010). Macronutrient (N,P,K) and redoximorphic metal (Fe, Mn) allocation in *Leersia. oryzoides* (Rice cutgrass) grown under different flood regimes. Water Air Soil Poll..

[343] Bostic E.M., White J.R. (2007). Soil phosphorus and vegetation influence on wetland phosphorus release after simulated drought. Soil Sci. Soc. Am. J..

[344] Richardson C.J. (1985). Mechanisms controlling phosphorus retention capacity in freshwater wetlands. Science.

[345] Smith D.R., Pappas E.A. (2007). Effect of ditch dredging on the fate of nutrients in deep drainage ditches of the Midwestern United States. J. Soil Water Conserv..

[346] Smith D.R., Huang C. (2010). Assessing nutrient transport following dredging of agricultural drainage ditches. Trans. ASABE.

[347] Arango C.P., Tank J.L., Schaller J.L., Royer T.V., Bernot M.J., David M.B. (2007). Benthic organic carbon influences denitrification in streams with high nitrate concentration. Freshwater Biol..

[348] Forshay K.J., Dodson S.I. (2011). Macrophyte presence is an indicator of enhanced denitrification and nitrification in sediments of a temperate restored agricultural stream. Hydrobiologia..

[349] Ullah S., Faulkner S.P. (2006). Denitrification potential of different land-use types in an agricultural watershed, lower Mississippi valley. Eco. Eng..

[350] Pierce S.C., Pezeshki S.R., Larsen D., Moore M.T. (2009). Hydrology and species-specific effects of *Bacopa.* monnieri and *Leersia. oryzoides* on soil and water chemistry. Ecohydrology.

[351] Veraart A.J., de Bruijne W.J.J., de Klein J.J.M., Peeters E.T.H.M., Scheffer M. (2011). Effects of aquatic vegetation type on denitrification. Biogeochemistry.

[352] DeBusk T.A., Peterson J.E., Reddy K.R. (1995). Use of aquatic and terrestrial plants for removing phosphorus from dairy wastewaters. Eco. Eng..

[353] Power M.E., Rainey W.E., Parker M.S., Sabo J.L., Smyth A., Khandwala S., Finlay J.C., McNeely F.C., Marsee K., Anderson C., Polis G.A., Power M.E., Huxel G. (2004). River to watershed subsidies in an old-growth conifer forest. Food Webs at the Landscape Level.

[354] Jackson J.K., Fisher S.G. (1986). Secondary production, emergence, and export of aquatic insects of a Sonoran Desert stream. Ecology.

[355] Richardson J.S., Zhang Y., Marczak L.B. (2010). Resource Subsides across the land-freshwater interface and responses in recipient communities. River Res. Appl..

[356] Nakano S., Murakami M. (2001). Reciprocal subsides: Dynamic interdependence between terrestrial and aquatic food webs. Proc. Nat. Acad. Sci. USA.

[357] Gratton C., Vander Zanden M.J. (2009). Flux of aquatic insect productivity to land: comparison of lentic and lotic ecosystems. Ecology.

[358] Lamberti G.A., Chaloner D.T., Hershey A.E. (2010). Linkages among aquatic ecosystems. J. N. Am. Benthol. Soc..

[359] Woods H.A., Fagan W.F., Elser J.J., Harrison J.F. (2004). Allometric and phylogenetic variation in insect phosphorus content. Funct. Ecol..

[360] Fagan W.F., Siemann E., Mitter C., Denno R.F., Huberty A.F., Woods H.A., Elser J.J. (2002). Nitrogen in insects: Implications for trophic complexity and species diversification. Am. Nat..

[361] Werner I., Markiewicz D.A., Goding K., Reece K., Moore M.T., Kröger R. (2010). Benthic macroinvertebrate communities in ephemeral agricultural drainage ditches of California’s Central Valley. Agricultural Drainage Ditches: Mitigation Wetlands for the 21^st^ Century.

[362] Blackburn M., Mazzacano C. (2012). Using Aquatic Macroinvertebrates As Indicators of Streamflow Duration: Washington and Idaho Indicators.

[363] Rabini C.F., Wallace G.S. The influence of flow variation on the ability to evaluate the biological health of headwater streams. Hydrology, Water Resources and Ecology in Headwaters. Proceedings of the HeadWater 1998 Conference.

[364] Williams D.D., Hynes H.B.N. (1976). The ecology of temporary streams I: The faunas of two Canadian streams. Int. Rev. Ges. Hydrobio..

[365] Feldman D.L., Farris J.L., Moore M.T., Cooper C.M., Moore M.T., Kroger R. (2010). A characterization of benthic macroinvertebrate communities in agricultural drainage ditches of the northeast Arkansas Delta, USA. Agricultural Drainage Ditches: Mitigation Wetlands for the 21^st^ Century.

[366] Shieh S., Ward J.V., Kondratieff B.C. (2002). Energy flow through macroinvertebrates in a polluted plains stream. J. N. Am. Benthol. Soc..

[367] Verdonschot R.C.M., Keizer-Vlek H.E., Verdonschot P.F.M. (2011). Biodiversity value of agricultural drainage ditches: a comparative analysis of the aquatic invertebrate fauna of ditches and small lakes. Aquat. Conserv. Mar. Freshwater Ecosys..

[368] Seale D. (1980). Influence of amphibian larvae on primary production, nutrient flux, and competition in a pond ecosystem. Ecology.

[369] Regester K.J., Whiles M.R., Lips K.R. (2008). Variation in the trophic basis of production and energy flow associated with emergence of larval salamander assemblages from forest ponds. Freshwater Biol..

[370] Regester K.J., Lips K.R., Whiles M.R. (2006). Energy flow and subsidies associated with the complex life cycle of ambystomatid salamanders in ponds and adjacent forest in southern Illinois. Oceologia.

[371] Relyea R.A. (2005). The impact of insecticides and herbicides on the biodiversity and productivity of aquatic communities. Eco. Appl..

[372] Manna R.M, Hyne R.V., Choung C.B., Wilson S.P. (2009). Amphibians and agricultural chemicals: Review of the risks in a complex environment. Environ. Pollut..

[373] Vanni M.J. (2002). Nutrient cycling by animals in freshwater ecosystems. Annu. Rev. Ecol. Syst..

[374] Small G.E., Helton A.M., Kazanci C. (2009). Can consumer stoichiometric regulation control nutrient spiraling in streams?. J. N. Am. Benthol. Soc..

[375] Nahlik A.M., Kentula M.E., Fennessy M.S., Landers D.H. (2012). Where is the consensus? A proposed foundation for moving ecosystem service concepts into practice. Eco. Econ..

